# Breast cancer cell–derived microRNA-155 suppresses tumor progression via enhancing immune cell recruitment and antitumor function

**DOI:** 10.1172/JCI157248

**Published:** 2022-10-03

**Authors:** Junfeng Wang, Quanyi Wang, Yinan Guan, Yulu Sun, Xiaozhi Wang, Kaylie Lively, Yuzhen Wang, Ming Luo, Julian A. Kim, E. Angela Murphy, Yongzhong Yao, Guoshuai Cai, Daping Fan

**Affiliations:** 1 Department of Cell Biology and Anatomy, University of South Carolina School of Medicine, Columbia, South Carolina, USA.; 2 Department of Life Science and Technology, China Pharmaceutical University, Nanjing, China.; 3 Department of General Surgery, Nanjing Drum Tower Hospital, Affiliated Hospital of Nanjing University Medical School, Nanjing, China.; 4 Department of Cardiology, First Affiliated Hospital of Nanjing Medical University, Nanjing, China.; 5 Department of Chemistry, Georgia State University, Atlanta, Georgia, USA.; 6 Prisma Health Surgical Oncology, Department of Surgery and; 7 Department of Pathology, Microbiology and Immunology, University of South Carolina School of Medicine, Columbia, South Carolina, USA.; 8 Department of Environmental Health Science, Arnold School of Public Health, University of South Carolina, Columbia, South Carolina, USA.

**Keywords:** Immunology, Breast cancer, Immunotherapy

## Abstract

Evidence suggests that increased microRNA-155 (miR-155) expression in immune cells enhances antitumor immune responses. However, given the reported association of miR-155 with tumorigenesis in various cancers, a debate is provoked on whether miR-155 is oncogenic or tumor suppressive. We aimed to interrogate the impact of tumor miR-155 expression, particularly that of cancer cell–derived miR-155, on antitumor immunity in breast cancer. We performed bioinformatic analysis of human breast cancer databases, murine experiments, and human specimen examination. We revealed that higher tumor miR-155 levels correlate with a favorable antitumor immune profile and better patient outcomes. Murine experiments demonstrated that miR-155 overexpression in breast cancer cells enhanced T cell influx, delayed tumor growth, and sensitized the tumors to immune checkpoint blockade (ICB) therapy. Mechanistically, miR-155 overexpression in breast cancer cells upregulated their CXCL9/10/11 production, which was mediated by SOCS1 inhibition and increased phosphorylated STAT1 (p-STAT1)/p-STAT3 ratios. We further found that serum miR-155 levels in breast cancer patients correlated with tumor miR-155 levels and tumor immune status. Our findings suggest that high serum and tumor miR-155 levels may be a favorable prognostic marker for breast cancer patients and that therapeutic elevation of miR-155 in breast tumors may improve the efficacy of ICB therapy via remodeling the antitumor immune landscape.

## Graphical abstract

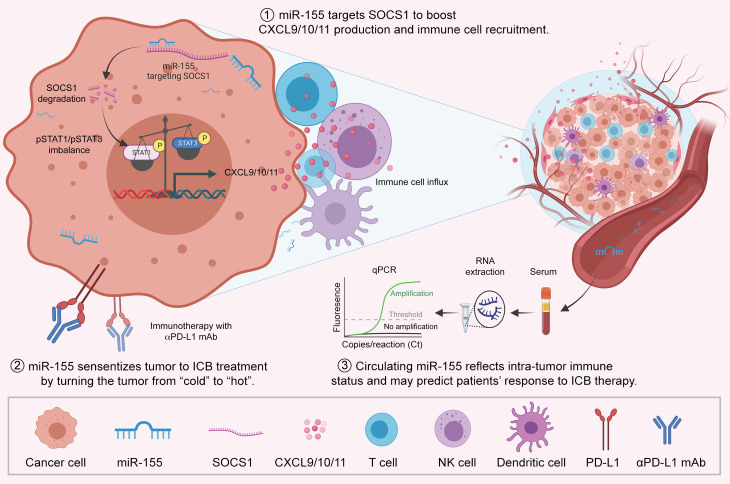

## Introduction

microRNA-155 (miR-155) was first identified as an oncomiR; it was found to promote carcinogenesis and disease progression of various hematological malignancies and solid tumors (1, 2). It is upregulated in cancers such as breast, liver, lung, pancreatic, and prostate (3–6). Nevertheless, some reports have shown that miR-155 upregulation in tumors is associated with improved overall survival of patients with several types of cancer, including breast cancer, colon cancer, and melanoma (7–9). To account for this discrepancy, we and others have shown that miR-155 may play a central role in innate and adaptive immune responses (10–16). We reported that miR-155 deficiency in macrophages and myeloid-derived suppressor cells (MDSCs) promotes tumor growth by exaggerating the immunosuppressive functions of these cells (12, 13). We also showed that miR-155 deficiency impairs DC maturation, cytokine secretion, migration toward tumor-draining lymph nodes, and ability to activate T cells, whereas miR-155 overexpression enhances these activities (14). Furthermore, using miR-155–overexpressing DCs, we generated a therapeutic vaccine that resulted in enhanced antitumor immunity against established breast tumors in mice, evidenced by increased intratumor effector T cells, suppressed tumor growth, and drastically reduced lung metastasis (15). miR-155 is also upregulated in activated lymphocytes and involved in T cell and B cell proliferation and maturation (17, 18). Depletion of miR-155 was intrinsically detrimental to the antitumor response of CD8^+^ cytotoxic T cells (19).

Since miR-155 is expressed in both immune cells and cancer cells in the tumors, an open question is whether miR-155 expression in breast tumors in general, and particularly in breast cancer cells, is pro- or antitumor. To answer this question, we performed bioinformatic analyses of human breast cancer databases, murine experiments, and human specimen examinations. We revealed that higher miR-155 levels in breast tumors are associated with favorable antitumor immune infiltration and better patient outcomes and that miR-155 expressed in breast cancer cells suppressed tumor progression by enhancing the recruitment of antitumor immune cells. Furthermore, we observed that miR-155–overexpressing tumors were more sensitive to immune checkpoint blockade (ICB) treatment in a mouse breast cancer model and that serum miR-155 abundance reflected intratumor miR-155 levels as well as the antitumor immune status of patients.

Our findings in this study are of translational and clinical value. Mirroring the immune status of breast tumors, circulating miR-155 levels may be used as a prognostic biomarker for breast cancer patients and as a predictive marker for their responsiveness to immunotherapeutic treatment. Moreover, strategies that enhance miR-155 expression in breast tumors may boost antitumor immunity and enhance the efficacy of ICB therapy.

## Results

### miR-155 expression levels in breast tumors are associated with disease progression.

**Figure 1:**
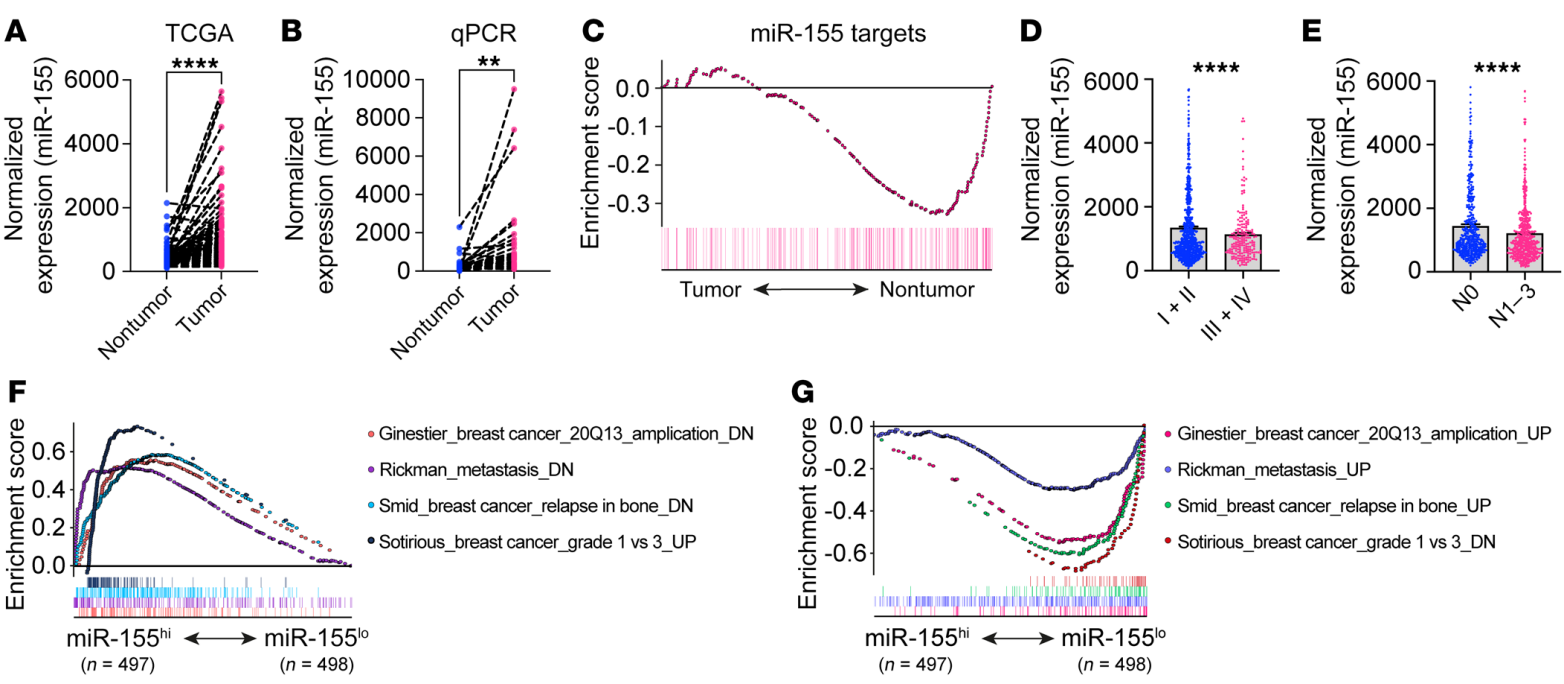
miR-155 expression levels in breast tumors are associated with disease progression. (**A**) Normalized miR-155 expression in paired human breast tumors and adjacent nontumor tissues by TCGA data. *n* = 99. (**B**) Relative miR-155 levels in paired human breast tumors and adjacent normal tissues by qPCR. *n* = 29. (**C**) GSEA analysis of TCGA data with respect to miR-155 target enrichment in tumor (*n* = 995) versus nontumor (*n* = 99) areas of human breast cancer patients. (**D**) Normalized miR-155 expression in breast tumors at different clinical stages. I + II, *n* = 732; III + IV, *n* = 204. (**E**) miR-155 levels in tumors of breast cancer patients with or without lymph node involvement. N0, *n* = 458; N1–3, *n* = 520. (**F**–**G**) Multi-GSEA analysis of tumor progression related gene signatures in miR-155^hi^ versus miR-155^lo^ tumors. All data are represented as mean ± SEM. **P < 0.01; ****P < 0.0001, paired (**A** and **B**) or unpaired (**C** and **D**) 2-tailed Student’s *t* test. DN, down.

To investigate the association of miR-155 with global gene expression and the clinical outcome of breast cancer patients, we retrieved and analyzed breast cancer data from miRNA-Seq and RNA-Seq and clinical information from The Cancer Genome Atlas (TCGA) Program (Supplemental Figure 1; supplemental material available online with this article; https://doi.org/10.1172/JCI157248DS1). After normalization and combination of the raw data, we found that miR-155 expression in human breast tumors was markedly higher than that in adjacent normal tissues (Supplemental Figure 2A). In 99 paired samples, we also observed that miR-155 levels were substantially higher in tumors compared with nontumor tissues from the same patients (Figure 1A). We confirmed this result by quantitative real-time PCR (qPCR) using freshly resected samples from a small cohort of breast cancer patients (Figure 1B and Supplemental Table 1). Aligning with these findings, we performed Gene Set Enrichment Analysis (GSEA) and found that miR-155 target genes were less enriched in tumors compared with nontumor tissues (Figure 1C), consistent with higher miR-155 levels in tumors and the degradation and/or depletion of miR-155 target genes.

To evaluate how miR-155 levels in tumors were associated with tumor progression, we analyzed relevant clinical information and associated the data with miR-155 expression in tumor tissues of breast cancer patients. The results showed that tumor miR-155 levels were higher in patients at early clinical stages (stages I and II; *n* = 732) than in those at advanced stages (stages III and IV; *n* = 204) (Figure 1D). In addition, in patients that had lymph node metastasis (N1–N3; *n* = 520), lower miR-155 levels were observed in their tumor tissues when compared with patients without lymph node involvement (N0; *n* = 458) (Figure 1E). In addition, GSEA results revealed that, in patients with miR-155 expression levels in the upper half (miR-155^hi^, *n* = 497), gene signatures associated with the downregulation of cancer amplification, metastasis, and relapse were extensively enriched, whereas the opposite was seen in patients with miR-155 expression levels in the lower half (miR-155^lo^, *n* = 498) (Figure 1, F and G).

### miR-155 levels in breast cancer tissues correlate with the outcome of patients.

**Figure 2:**
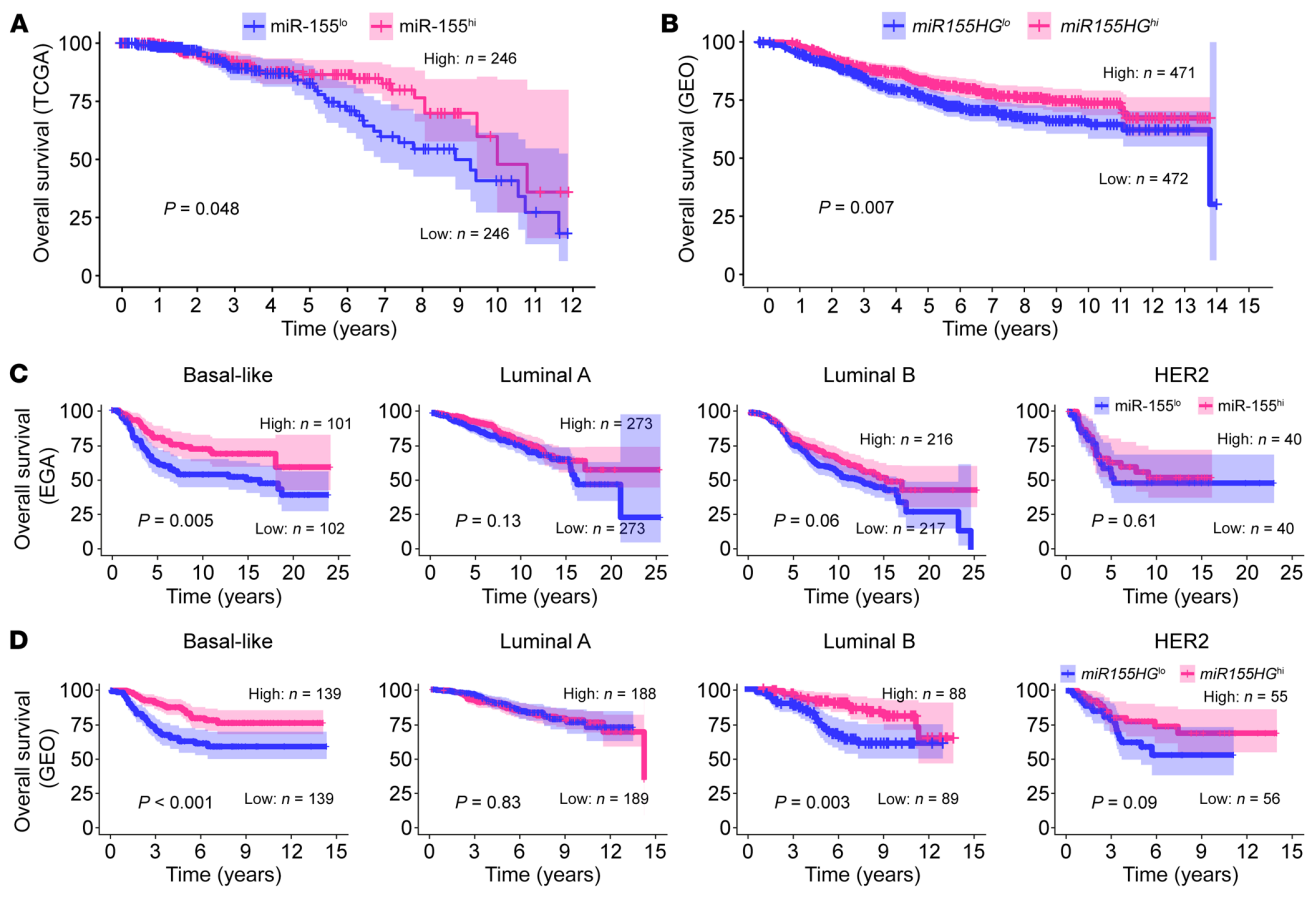
Higher miR-155 levels in human breast tumors are associated with better patient outcome. (**A**) Overall survival of breast cancer patients from TCGA database with high or low levels of miR-155 expression. Patients were divided into 2 groups according to the upper and lower quartiles of normalized miR-155 levels in tumors. *n* = 246 in each group. (**B**) Overall survival of breast cancer patients from pooled GEO database with high and low levels of *miR155HG* expression. (**C** and **D**) Overall survival of breast cancer patients with different molecular classifications and miR-155 expression levels from EGA (**C**) and GEO (**D**) data sets. For **B**–**D**, patients were divided into 2 groups according to the median value of miR-155 or *miR155HG* levels in tumors. The curves comparison with the log-rank (Mantel-Cox) test revealed statistically significant differences as shown in graphs. HG, host gene.

Based on the miR-155 expression levels in the tumor tissues, we categorized the upper and lower quartiles of patients in TCGA database as miR-155^hi^ and miR-155^lo^, with 246 patients in each group. Compared with miR-155^lo^ patients, miR-155^hi^ patients exhibited an extended survival time (Figure 2A). Multivariate Cox’s proportional hazards analysis demonstrated that high miR-155 expression in tumors was a protective factor for breast cancer patients (HR = 0.724, *P* = 0.028) (Supplemental Table 2), suggesting that miR-155 can be used as an independent prognostic factor for breast cancer patients. To confirm this result, we analyzed the relationship between miR-155 and miR-155 host gene (*miR155HG*) expression levels and the overall survival of different cohorts of breast cancer patients. The data of the European Genome-Phenome Archive (EGA) cohort showed that patients with higher miR-155 levels had extended survival time, although statistical significance was not reached (*P* = 0.34) (Supplemental Figure 2B). Notably, the results of meta-analysis of multiple Gene Expression Omnibus (GEO) data sets, which were retrieved from a Kaplan-Meier (KM) plotter, support a positive relationship between *miR155HG* expression levels and the survival rates of breast cancer patients (Figure 2B) To further interrogate the relationship between miR-155 levels and the prognosis of patients with cancers of differing molecular classifications, we generated KM survival curves of various breast cancer subtypes with different miR-155 expression levels; the results showed that in EGA and GEO cohorts, miR-155 or *miR155HG* expression levels were positively associated with the outcome of breast cancer patients, regardless of molecular subtype, although the association was not statistically significant in the luminal A and HER2 patients (Figure 2, C and D). By analyzing TCGA patient groups based on the median value of their tumor miR-155 expression levels, we also found a significant association between miR-155 expression levels and overall survival of luminal B patients, and similar trends were also observed in basal-like and HER2-type breast cancer patients, although the associations were not statistically significant due to the small sample size (Supplemental Figure 2C).

Collectively, these data suggest that miR-155 expression levels in breast tumors are inversely associated with breast cancer progression and positively correlated with better patient outcome. Based on our recent findings indicating that miR-155 is a central regulator of antitumor immunity (12–15), we speculate that patients who have high tumoral miR-155 expression may have enhanced antitumor immune responses.

### Higher miR-155 expression defines a better antitumor immune profile in human breast tumors.

**Figure 3:**
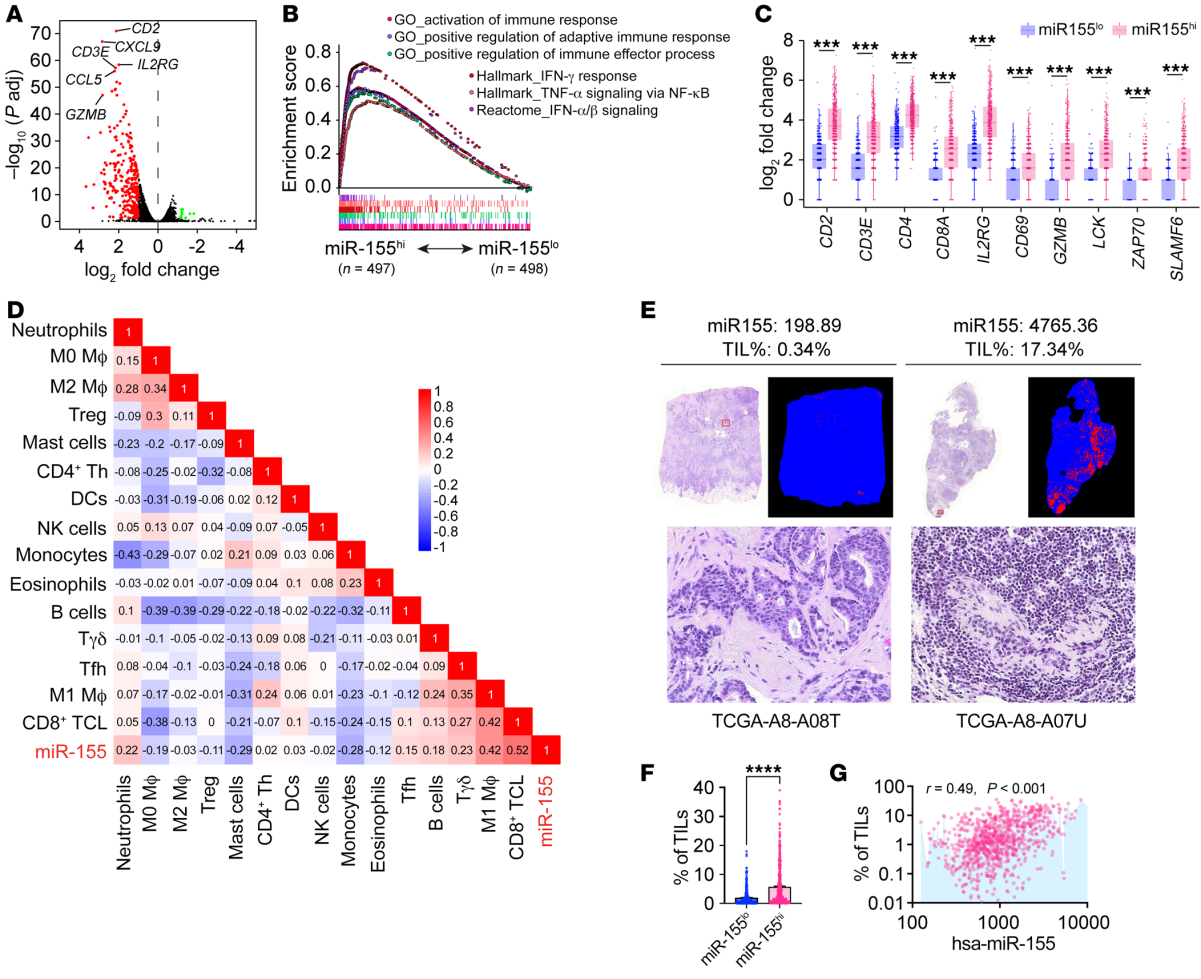
miR-155 expression levels in breast tumors are positively correlated with antitumor immunity. (**A**) Volcano plot for the DEGs in miR-155^hi^ versus miR-155^lo^ tumors. *P* adj, adjusted value. (**B**) Multi-GSEA analysis of immune-related gene signatures in miR-155^hi^ versus miR-155^lo^ tumors. (**C**) Box plots comparing T cell–associated gene expression between miR-155^hi^ (*n* = 497) and miR-155^lo^ (*n* = 498) tumors. (**D**) Correlations of normalized miR-155 expression with predicted immune cell fractions in breast cancer tumors. *n* = 995. (**E**) Representative H&E staining and computational staining images of breast cancer tumors from TCGA, which were retrieved from the CANCER Digital Slide Archive and TCIA, respectively. Normalized miR-155 expression and TIL percentage values are shown above corresponding images. (**F**) Quantification of estimated TIL proportions in miR-155^hi^ and miR-155^lo^ breast cancer tumors. *n* = 432 per group. (**G**) Correlations of miR-155 levels with the percentages of TILs in breast cancer tumor tissues. *n* = 864. (**C**) Wilcoxon’s rank sum test was carried out to compare T cell activation–related gene expression between miR-155^hi^ and miR-155^lo^ breast cancer tumors. ***P < 0.001. (**D** and **G**) *P* and *r* value were calculated based on Pearson’s correlation analysis. (**F**) Statistical significance was assessed using unpaired, 2-tailed Student’s *t* test, and all data are represented as mean ± SEM. ****P < 0.0001.

To investigate whether high miR-155 expression in tumors was associated with an enhanced antitumor immune response, we first analyzed differentially expressed genes (DEGs) in miR-155^hi^ versus miR-155^lo^ tumors from TCGA database. Based on the preset criteria of log_2_ fold change greater than or equal to 1 and adjusted *P* value of less than 0.05, 293 out of 12,885 genes were shown to be differentially expressed, including 283 genes that were upregulated and 10 genes that were downregulated in miR-155^hi^ tumors (Figure 3A). The DEGs with a log_2_ fold change of at least 2.0 (*n* = 64) are shown in Supplemental Table 3. Functional enrichment of DEGs was performed by Kyoto Encyclopedia of Genes and Genomes (KEGG) pathway analysis and Gene Ontology (GO) term analysis. KEGG analysis revealed that the pathways enriched in miR-155^hi^ tumors were mostly immune related (Supplemental Figure 3A). Consistently, the expression levels of antitumor gene clusters, including genes involved in lymphocyte activation and antigen processing and presentation, were markedly enriched in miR-155^hi^ tumors (Supplemental Figure 3B). These results were confirmed by GSEA analysis showing that the immune-response signatures, such as lymphocyte activation and IFN signaling, were strongly enriched in miR-155^hi^ tumors (Figure 3B). Specifically, the expression of T cell functional molecules was dramatically upregulated in the miR-155^hi^ tumors (Figure 3C), indicating an augmented antitumor immunity within these tumors.

To further confirm the relationship between miR-155 expression and tumor immune profiles, we next applied the CIBERSORTx algorithm (20), which deconvolved the genomic data to estimate the fraction of immune cells in both miR-155^hi^ and miR-155^lo^ tumor tissues. The correlations between miR-155 expression and total immune cell proportions were generated using the R script. The results showed that miR-155 expression levels in tumors were positively correlated with multiple antitumoral immune cell types, including CD8^+^ T cells and M1 macrophages; the results also showed that miR-155 levels were negatively associated with the frequencies of protumoral immune cell types, such as Tregs and M2 macrophages (Figure 3D and Supplemental Figure 4). Consistently, using another convolutional neural network–based atlas developed by The Cancer Image Archive (TCIA) (21), we found that the estimated proportion of tumor-infiltrating lymphocytes (TILs) was positively associated with miR-155 levels in human breast tumors (Figure 3, E–G). Together, these data suggest that increased miR-155 levels are positively associated with enhanced innate and adaptive immunity in human breast tumors.

### Overexpression of miR-155 in breast cancer cells delays tumor growth and increases antitumor immune infiltration.

**Figure 4:**
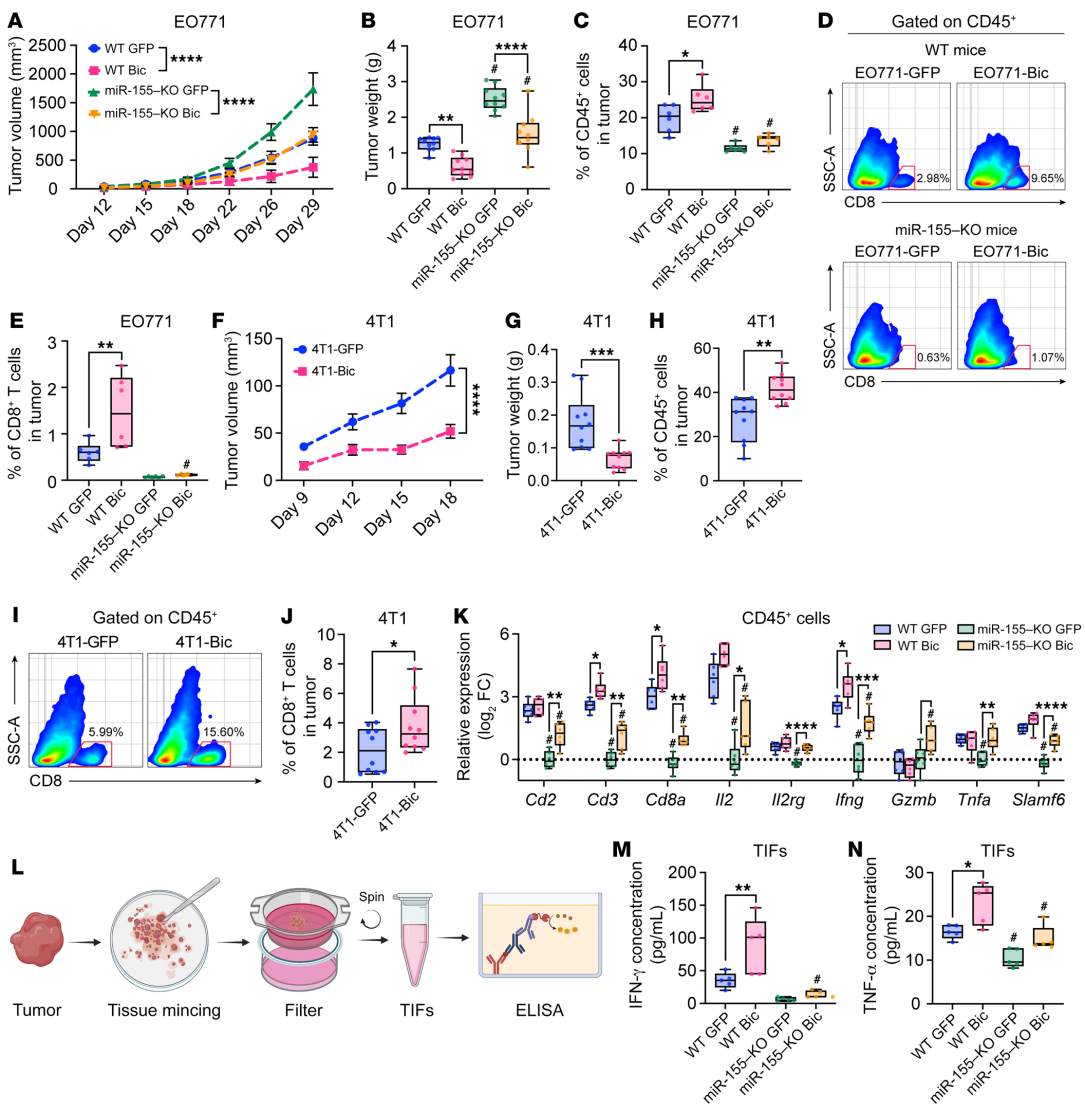
Forced miR-155 overexpression inhibits tumor growth by increasing immune cell influx. (**A**) EO771-GFP and EO771-Bic tumor growth curves in WT or miR-155–KO mice. *n* = 10–20 per group. (**B**) EO771 tumor weight 29 days after tumor inoculation. *n* = 10 per group. (**C**) Frequency of tumor-infiltrating CD45^+^ leukocytes by flow cytometry. *n* = 6 per group. (**D**) Representative pseudo color images from 6 samples of each group showing the frequency of CD8^+^ T cells gating from CD45^+^ cells. (**E**) Quantified percentage of CD8^+^ T cells in EO771 tumors. (**F**) 4T1-GFP and 4T1-Bic tumor growth curves in BALB/c mice. 4T1 tumor weight (**G**) and CD45^+^ immune cell percentages (**H**) 19 days after tumor inoculation. (**I**) Representative pseudo color images showing the frequency of CD8^+^ T cells gating from CD45^+^ cells. (**J**) Quantified percentage of CD8^+^ T cells in 4T1 tumors. *n* = 10 per group (**F**–**J**). (**K**) T cell activation–related gene expression in sorted tumor-infiltrating CD45^+^ cells by qPCR. *n* = 6 per group. (**L**) Schematic image illustrating the procedure of TIF collection from tumor tissue and ELISA. IFN-γ (**M**) and TNF-α (**N**) protein concentrations in TIFs. Statistical analysis of **A** and **F** was performed using 2-way ANOVA followed by Tukey’s test. Statistical significance was assessed using 2-tailed Student’s *t* test for comparing 2 groups (**G**, **H** and **J**) and 1-way ANOVA followed by Tukey’s post hoc test for multiple groups (**B**, **C**, **E**, **K**, **M**, and **N**). All data are represented as mean ± SEM. ^#^*P* < 0.05, compared with WT counterparts; *P < 0.05, **P < 0.01, ***P < 0.001, ****P < 0.0001.

miR-155 in tumors is derived from both cancer cells and stroma cells, including immune cells. While we and others showed the antitumor role of immune cell miR-155 (10–19), the role of cancer cell–derived miR-155 is more elusive and controversial (5, 8). To investigate the direct impact of cancer cell–derived miR-155 on tumor progression and tumor immune infiltration, we established a B cell integration cluster (Bic, miR-155 gene) overexpressing breast cancer cell lines (EO771-Bic, 4T1-Bic, and AT-3-Bic) via lentiviral transduction. These cells express 15- to 60-fold higher miR-155 than control lentiviral transduced cells (EO771-GFP, 4T1-GFP, and AT-3-GFP) (Supplemental Figure 5, A–C). miR-155–overexpressing breast cancer cells exhibited proliferative capacities comparable to those of the control cells in vitro (Supplemental Figure 5, D–F) as well as similar sensitivities to doxorubicin (Supplemental Figure 5, G and H). Despite these, the growth rates of the tumors with miR-155–overexpressing EO771 cells were significantly delayed compared with those of EO771-GFP counterparts in C57BL/6 WT mice as well as in miR-155–KO mice (Figure 4, A and B). Consistent with our previous report (14), host miR-155 deficiency dramatically accelerated EO771 tumor growth.

We next examined the immune profiles in EO771-Bic and EO771-GFP tumors using flow cytometry. The results showed the frequencies of CD45^+^ immune cells were significantly increased in EO771-Bic tumors compared with EO771-GFP tumors (Supplemental Figure 6A and Figure 4C). Specifically, overexpression of miR-155 in EO771 breast cancer cells increased the presence of antitumor immune cells, including DCs, helper T cells, cytotoxic T cells, and tumoricidal NK cells (Figure 4, D and E, and Supplemental Figure 6B). Consistently, in the 4T1 breast cancer model, overexpressing miR-155 in cancer cells also significantly inhibited tumor growth (Figure 4, F and G), which was accompanied by increased tumor-infiltrating immune cells (Figure 4, H–J) compared with the control counterparts. Additionally, the T cells in EO771-Bic tumors were detected with enhanced proliferative capacity by an in vivo BrdU incorporation assay (Supplemental Figure 6C). Moreover, we discovered an increased number of apoptotic cancer cells in EO771-Bic tumors by flow cytometry analysis and TUNEL assay (Supplemental Figure 6, D and E). Concerning immune cell composition, we detected a dramatically lower level of immune infiltration in miR-155–KO mice compared with WT mice, which can be explained by their intrinsic defects in CCR5/CXCR3 expression levels (Supplemental Figure 7, A and B).

Our results consistently showed that T cell activation–related genes were drastically upregulated in EO771-Bic tumor-infiltrating CD45^+^ leukocytes isolated from both WT and miR-155–KO mice (Supplemental Figure 8 and Figure 4K). Furthermore, IFN-γ and TNF-α were markedly enriched in the tumor interstitial fluids (TIFs) retrieved from EO771-Bic tumors compared with those from EO771-GFP tumors (Figure 4, L–N). Taken together, these results, particularly those from the miR-155–KO mice, indicate that miR-155 produced by breast cancer cells enhances immune cell influx and antitumor capacity, resulting in substantial tumor suppression.

### miR-155 overexpression in cancer cells enhances immune cell influx by increasing the production of chemoattractants via suppressing SOCS1 and tilting the p-STAT1/p-STAT3 balance.

**Figure 5:**
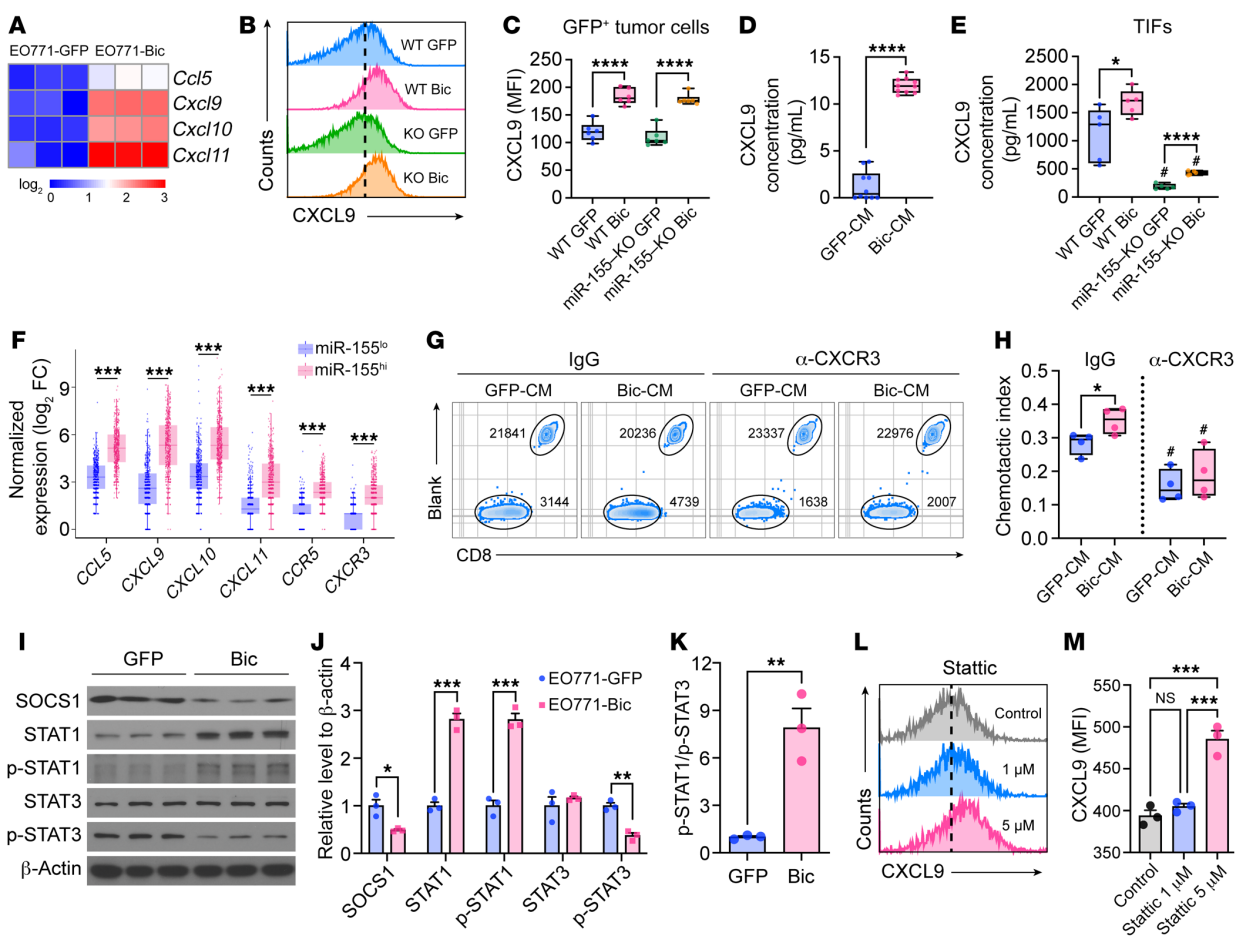
miR-155 overexpression enhances T cell recruitment by upregulating CCL5 and CXCL9/10/11 expression via tilting the p-STAT1/p-STAT3 ratio. (**A**) *Ccl5* and *Cxcl9/10/11* expression by qPCR. *n* = 3 per group. (**B** and **C**) Intracellular CXCL9 expression in EO771-GFP/Bic cells retrieved from tumor tissue (GFP^+^ cells) by flow cytometry. Representative histograms (**B**) and quantified MFI of CXCL9 (**C**) are shown. *n* = 6 per group. CXCL9 concentration in cell culture media (**D**) and TIFs (**E**) by ELISA. *n* = 6 per group. (**F**) Expression of T cell recruitment–related genes in miR-155^hi^ (*n* = 497) and miR-155^lo^ (*n* = 498) human breast cancer. (**G**) In vitro T cell migration toward EO771-GFP/Bic cell culture media. Representative zebra plots showing the number of T cells and beads by flow cytometry. (**H**) Chemotactic index of **G** was calculated based on estimated cell numbers using counting beads. *n* = 4 per group. (**I**) Representative Western blotting bands showing SOCS1 and STAT1/STAT3 levels in EO771-GFP or EO771-Bic cells. (**J**) Blots shown in **I** were quantified relative to β-actin expression. *n* = 3 per group. For samples run on different gels, separate loading controls are provided in the supplemental material. (**K**) p-STAT1 to p-STAT3 ratio based on band intensity. *n* = 3 per group. (**L** and **M**) Intracellular CXCL9 expression in EO771 parental cells 24 hours after STAT3 inhibitor (Stattic) treatment. Representative histograms (**L**) and quantified MFI of CXCL9 (**M**) are shown. *n* = 3 per group. Statistical significance in all panels except **F** was assessed using the unpaired, 2-tailed Student’s *t* test. Statistical results shown in **F** were carried out by Wilcoxon’s rank sum test. All data are represented as mean ± SEM. ^#^*P* < 0.05 compared with WT counterparts; *P < 0.05, **P < 0.01, ***P < 0.001, ****P < 0.0001.

To corroborate the above finding that miR-155 overexpression in breast cancer cells helps flood the tumor with antitumor immune cells, we performed an unbiased multiplex proinflammatory chemokine panel assay to determine the secretome difference between EO771-GFP and EO771-Bic cells. Among 13 types of chemokines tested, we found the concentrations of key chemoattractants for T cell recruitment, including CCL5, CXCL9, and CXCL10, were significantly enriched in EO771-Bic cell conditioned medium (Bic-CM) compared with that in EO771-GFP cell conditioned medium (GFP-CM) (Supplemental Figure 9, A–C). We next confirmed that miR-155 overexpression upregulated *Ccl5* and *Cxcl9/10/11* expression in murine breast cancer cell lines (Figure 5A and Supplemental Figure 10, A and B) using qPCR. Given that the differential expression of *Cxcl9/10/11* is the highest between GFP and Bic tumor cells and CXCL9/10/11 share similar regulatory mechanisms and bind to the same receptor, CXCR3 (22), we chose CXCL9 as the representative to investigate how breast cancer cell–derived miR-155 promotes T cell recruitment via upregulating this chemoattractant. We found that CXCL9 protein levels were remarkably upregulated in miR-155–overexpressing cancer cells in the tumors (Figure 5, B and C) and in cell culture (Supplemental Figure 10, C and D) as well as in miR-155–overexpressing cell culture medium (Figure 5D) and EO771-Bic TIF (Figure 5E). Consistent with the murine tumor model, we found in TCGA database that the expression of T cell recruitment–related genes was substantially increased in miR-155^hi^ human breast tumors (Figure 5F) and positively correlated with tumor miR-155 levels (Supplemental Figure 10E).

To confirm that miR-155–overexpressing breast cancer cells attract more activated T cells, we performed an in vitro T cell migration assay using OVA peptide 257–264 to stimulate OT-I CD8^+^ T cells, which express high levels of CXCR3 (23). As expected, the Bic-CM was more potent in attracting activated T cells than GFP-CM, and T cell migration toward GFP-CM and Bic-CM was significantly attenuated and the difference diminished by CXCR3 blockade (Figure 5, G and H), suggesting the CXCL9/10/11-CXCR3 axis plays an essential role in tumor-intrinsic miR-155–mediated T cell influx to the tumor.

SOCS1 has been identified as an important miR-155 target (14) and an inhibitor of cytokine-induced signaling that acts via the JAK/STAT pathway (24). Among STAT proteins, STAT1 and STAT3 are reported to regulate the expression of CXCL9/10/11 in myeloid cells (25, 26) and play opposing roles in directing cellular activities (26, 27). To determine whether miR-155 upregulates CXCL9/10/11 expression by targeting SOCS1 and thereby altering downstream STATs, we performed Western blot analysis. We found markedly reduced SOCS1 levels accompanied by increased p-STAT1 and STAT1, decreased p-STAT3, and thus an increased p-STAT1/p-STAT3 ratio in miR-155–overexpressing EO771, 4T1, and AT-3 tumor cells compared with control cells (Figure 5, I–K, and Supplemental Figure 11, A–C). Importantly, we obtained consistent results using human primary breast cancer cells transduced with lentiviruses to introduce miR-155 overexpression. Specifically, we found that miR-155 overexpression in human primary breast cancer cells significantly increased *Ccl5* and *Cxcl10/11* expression (Supplemental Figure 12, A–E). In addition, Western blot analysis showed decreased SOCS1, but increased phosphorylated STAT1 (p-STAT1)/p-STAT3 ratios in miR-155–overexpressing primary cancer cells compared with controls (Supplemental Figure 12, F–H). To further confirm that SOCS1 is the miR-155 target that regulates CXCL9/10/11 expression via regulating p-STAT1/p-STAT3 balance, we generated SOCS1 knockdown EO771 cells using siRNA transfection (Supplemental Figure 13A). We found that the EO771 cells with reduced SOCS1 expression displayed a phenotype similar to that of miR-155–overexpressing cells, including enhanced *Cxcl9* and *Cxcl11* expression (Supplemental Figure 13, B and C), as well as an increased p-STAT1/p-STAT3 ratio (Supplemental Figure 13, D–F).

These data indicate that the increased p-STAT1/p-STAT3 ratio may have led to increased CXCL9/10/11 expression in miR-155–overexpressing breast cancer cells. Indeed, STAT3 inhibition by Stattic phenocopied miR-155 overexpression and enhanced CXCL9 production in breast cancer cells (Figure 5, L and M, and Supplemental Figure 11D). Taken together, these results suggest that miR-155 in breast cancer cells enhances CXCL9/10/11 expression by suppressing SOCS1 expression and tilting the p-STAT1/p-STAT3 ratio, leading to the recruitment of effective T cells to the tumor site and subsequently an improved antitumor immune response.

### miR155 deficiency promotes tumor progression by impairing immune cell recruitment.

**Figure 6:**
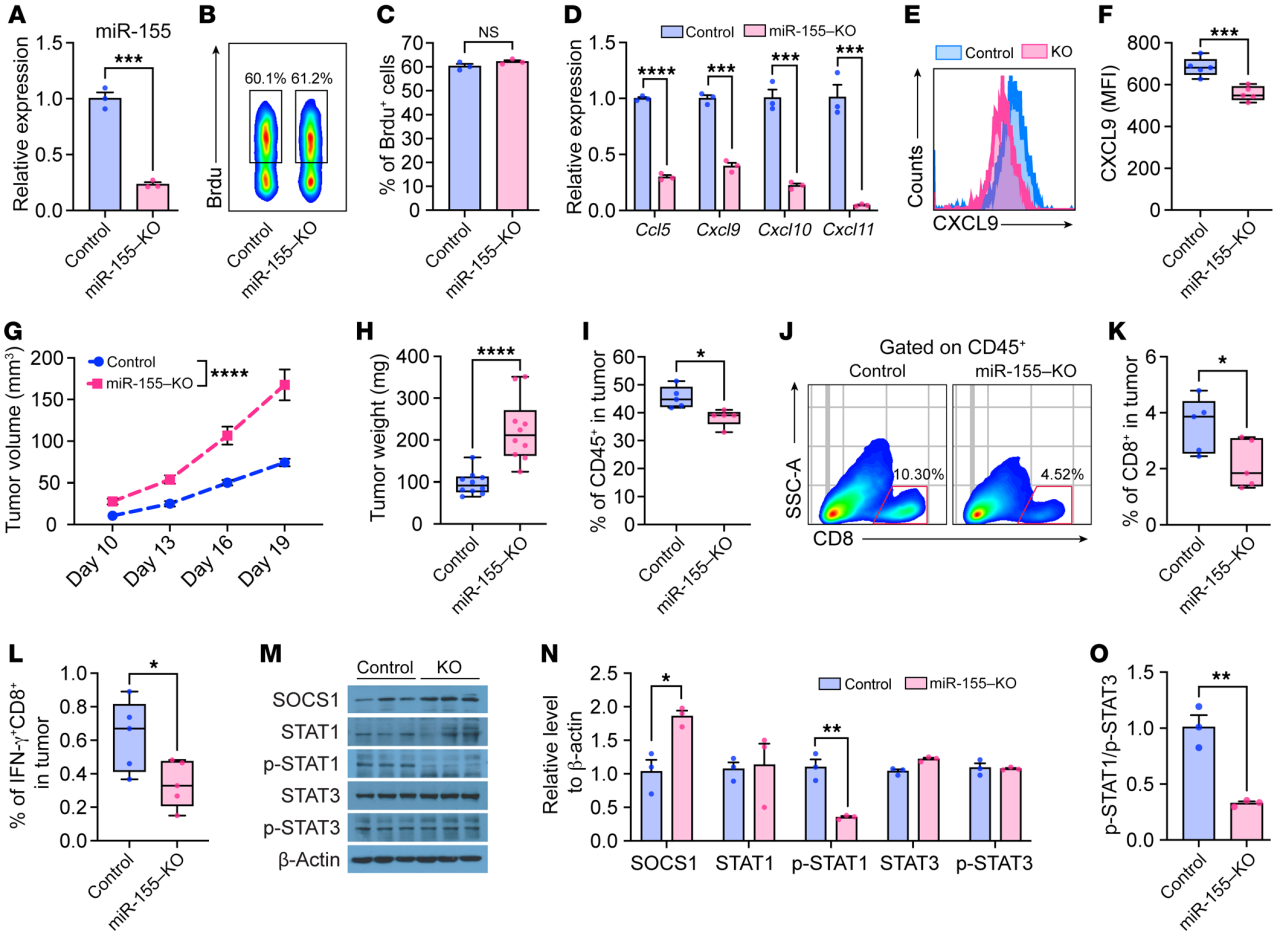
miR-155 KO in EO771 cells promotes tumor growth by impairing antitumor immune infiltration. (**A**) Validation of miR-155 expression in miR-155–KO EO771 cells by qPCR. *n* = 3 per group. Representative pseudo color image (**B**) and quantified data (**C**) showing percentage of Brdu^+^ control and miR-155–KO EO771 cells. *n* = 3 per group. (**D**) *Ccl5* and *Cxcl9/10/11* expression in control and miR-155–KO EO771 cells by qPCR. *n* = 3 per group. Representative histograms (**E**) and quantified MFI (**F**) of intracellular CXCL9 in control and miR-155–KO EO771 cells by flow cytometry. *n* = 5 per group. (**G**) EO771-control and EO771-miR-155–KO tumor growth curves in WT mice. *n* = 10 per group. (**H**) Tumor weight 19 days after tumor inoculation. *n* = 10 per group. (**I**) Frequency of tumor-infiltrating CD45^+^ leukocytes by flow cytometry. *n* = 5 per group. (**J**) Representative pseudo color images from 5 samples of each group showing the frequency of CD8^+^ T cells gating from CD45^+^ cells. Quantified percentage of CD8^+^ (**K**) and IFN-γ^+^CD8^+^ (**L**) T cells in tumors. (**M**) Representative Western blotting bands showing SOCS1 protein and STAT1/STAT3 protein and phosphorylation levels in EO771-control and EO771-miR-155–KO cells. For samples run on different gels, separate loading controls are provided in the supplemental material. (**N**) Blots shown in **M** were quantified relative to β-actin expression. *n* = 3 per group. (**O**) The ratio of p-STAT1 to p-STAT3 in EO771-control and EO771-miR-155–KO cells based on band intensity. *n* = 3 per group. Statistical significance in all figures was assessed using the unpaired, 2-tailed Student’s *t* test. All data are represented as mean ± SEM. *P < 0.05, **P < 0.01, ***P < 0.001, ****P < 0.0001.

To further verify the above findings, we generated miR-155–KO EO771 cells using the CRISPR-Cas9 genomic editing system. miR-155 levels in miR-155–KO cells were decreased by about 75 % (Figure 6A) without affecting cell proliferation in vitro (Figure 6, B and C). We speculate that miR-155 was not completely eliminated in the cells due to the endocytosis of miR-155 in FBS contained in the culture medium, as the sequence of miR-155 is highly conserved among many species (28). We obtained results opposite of those we found in EO771-Bic cells, including significantly reduced *Ccl5* and *Cxcl9/10/11* expression in miR-155–KO EO771 cells (Figure 6D). In addition, we also detected decreased intracellular CXCL9 protein levels via flow cytometry (Figure 6, E and F). Importantly, miR-155–KO EO771 tumors grew faster in vivo compared with their control counterparts (Figure 6, G and H). Immune profile analysis displayed reduced immune cells (Figure 6I), including reduction of antitumor CD8^+^ T cell (Figure 6, J–L) accumulation in tumor tissues. Mechanistically, we detected an increase in SOCS1, but a decrease in the p-STAT1/p-STAT3 ratio in miR-155–KO EO771 cells (Figure 6, M–O). Taken together, our in vitro and in vivo data using miR-155–KO tumor cells further confirms the antitumor role of endogenous miR-155 in regulating antitumor immune response by targeting SOCS1 and altering its downstream p-STAT1/p-STAT3 balance.

### miR-155^hi^ tumors have elevated expression of immuno-break molecules.

Emerging evidence has revealed that increased expression of immunosuppressive molecules is concomitant with an activated immune response. This negative feedback loop is essential for maintaining normal immune responses and limiting T cell activity to protect normal cells during chronic inflammation (29, 30). However, tumors may circumvent T cell–mediated cytotoxicity by expressing immunosuppressive molecules on both cancer cells and tumor-infiltrating immune cells, resulting in the inhibition of immune-mediated cancer cell death (30).

**Figure 7:**
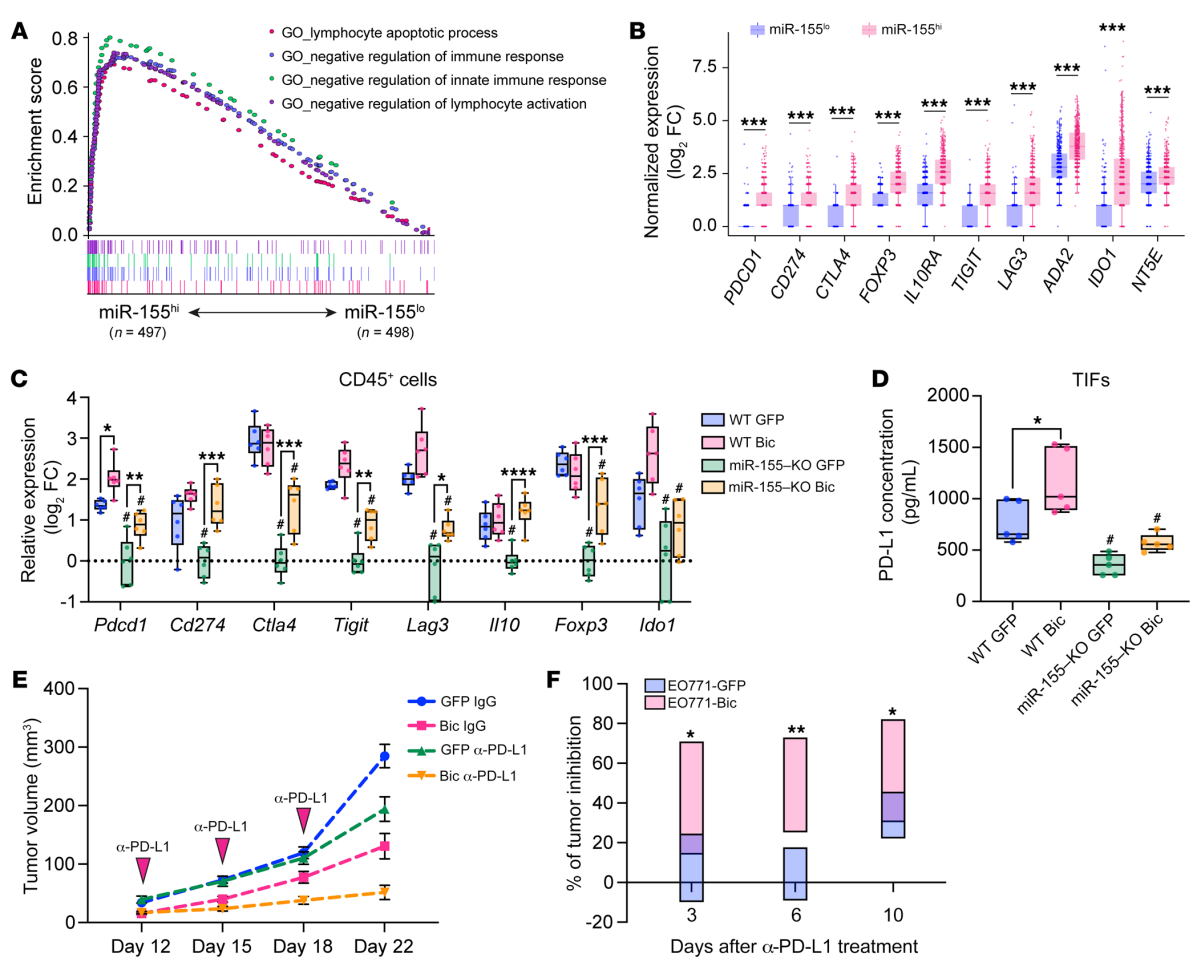
High miR-155 expression increases the level of immuno-break molecules and improves the tumor response to immunotherapy. (**A**) Multi-GSEA analysis showing the enrichment of negative immune response signatures in miR-155^hi^ (*n* = 497) and miR-155^lo^ (*n* = 498) human breast cancer tumors. (**B**) Box plot showing T cell exhaustive and immunosuppressive genes in miR-155^hi^ (*n* = 497) and miR-155^lo^ (*n* = 498) tumors. (**C**) Relative expression of T cell exhaustive and immunosuppressive genes in sorted tumor-infiltrating leukocytes from tumor-bearing mice. *n* = 6 per group. (**D**) Soluble PD-L1 concentration in TILs by ELISA. *n* = 6 per group. (**E**) Tumor growth curves of EO771-GFP or EO771-Bic tumors of mice treated with anti–PD-L1 mAbs. IgG2b was applied as isotype control. *n* = 7–10 per group. (**F**) Percentage of tumor inhibition at various time points after anti–PD-L1 mAb treatment. *n* = 7–9 per group. Statistical significance was assessed using 2-tailed Student’s *t* test for comparing 2 groups (**F**) and 1-way ANOVA followed by Tukey’s post hoc test for multiple groups (**C** and **D**). All data are represented as mean ± SEM. ^#^*P* < 0.05 compared with WT counterparts; *P < 0.05, **P < 0.01, ***P < 0.001, ****P < 0.0001.

GSEA analysis of TCGA database showed that the negative regulators of immune response and lymphocyte apoptotic processes were highly enriched in miR-155^hi^ human breast tumors (Figure 7A). Specifically, the expression of hallmark immunosuppressive genes, including *PDCD1* (PD1), *CD274* (PD-L1), *CTLA4*, and *FOXP3*, was drastically upregulated in miR-155^hi^ tumors (Figure 7B). Consistent with TCGA data, we found that in CD45^+^ leukocytes isolated from EO771-Bic tumors in both WT and miR-155–KO mice, the overall expression of main checkpoint molecules was substantially increased (Figure 7C). Furthermore, the concentrations of soluble PD-L1 in the TIFs harvested from Bic tumors were significantly higher than those from control GFP tumors (Figure 7D). In addition, not only the expression of PD-L1 on miR-155–overexpressing human primary and murine breast cancer cells was upregulated (Supplemental Figure 14, A–F), but also the expression of PD-L1 on tumor-associated macrophages (TAMs) was significantly upregulated in Bic tumors compared with that in control GFP tumors (Supplemental Figure 14, G–J). These data suggest that the enhanced antitumor immunity elicited by cancer cell miR-155 overexpression also triggers immunosuppressive pathways in breast tumors, which may set a stage for ICB therapy.

### Tumors with miR-155–overexpressing cancer cells display an improved response to immunotherapy and elicit a stronger immunological memory.

The elevated expression of immuno-break molecules in miR-155^hi^ tumors prompted us to explore whether elevated miR-155 levels in breast cancer cells could sensitize the tumors to ICB therapy. We treated established EO771-GFP and EO771-Bic tumors with anti–PD-L1 mAbs and observed that EO771-Bic tumors were more sensitive throughout the treatment cycle, as determined by percentage of tumor inhibition (Figure 7, E and F).

Abundant evidence indicates that exosomes carry bioactive miRNAs that can shuttle between tumor cells and other types of cells in the TME, therefore affecting many aspects of tumor development, including immune cell activities (31, 32). Based on recent findings indicating that miR-155 is antitumoral in multiple immune cells (2, 8, 10–15, 18, 19), we hypothesized that the exosomes containing miR-155 produced by tumor cells may directly facilitate immune cell activation in the TME. To address this question, we purified exosomes from EO771-GFP and EO771-Bic tumor–conditioned media by differential centrifugation (Supplemental Figure 15A). miR-155 levels in Bic cell–derived exosomes (Bic-Exo) were about 150-fold higher than those in GFP cell–derived exosomes (GFP-Exo) (Supplemental Figure 15B). To investigate whether tumor-derived exosomal miR-155 affects antitumor immunity in vivo, we injected a single dose of 50 μg of each exosome type i.v. into EO771 tumor–bearing mice (Supplemental Figure 15C). Three days later, we analyzed the immune profile within tumors and tumor-draining lymph nodes and found that Bic-Exo administration elicited an enhanced antitumor immune response, characterized by augmented immune response in tumor-draining lymph nodes (Supplemental Figure 15, D–F) as well as increased presence of overall CD45^+^ immune cells (Supplemental Figure 15G) and cytotoxic CD8^+^ T cells in tumor tissues (Supplemental Figure 15, H–J).

To explore whether miR-155 overexpression in cancer cells elicits immune memory in tumor-bearing mice, we surgically removed EO771-GFP or EO771-Bic tumors 20 days after inoculation and rechallenged the same mice with parental EO771 cells in the contralateral mammary fat pad and with B16-F10 melanoma cells on the back and then monitored the growth of the new EO771 and B16-F10 tumors (Supplemental Figure 16A). Some naive mice were also challenged with parental EO771 and B16-F10 tumors as control. The results showed that the mice that previously had EO771-GFP tumors displayed a modest increase in tumor-specific immune memory, which curbed the growth of the reinoculated breast tumors compared with those in the control naive mice, but without statistical significance (*P* = 0.12 on day 17); however, melanoma progression in these mice was observed to be accelerated. Interestingly, the mice that previously had EO771-Bic tumors almost completely rejected both newly transplanted EO771 tumors and B16-F10 melanomas (Supplemental Figure 16, B and C), and their survival time was dramatically extended (Supplemental Figure 16D). These data suggest that to a certain extent, EO771-GFP tumors established immunological memory to the same cancer type, but the immunosuppressive metabolites released by invasive tumors might also compromise systemic immune function, which would then favor the development of a different type of tumor.

### Serum miR-155 level mirrors the immune status of breast tumors.

**Figure 8:**
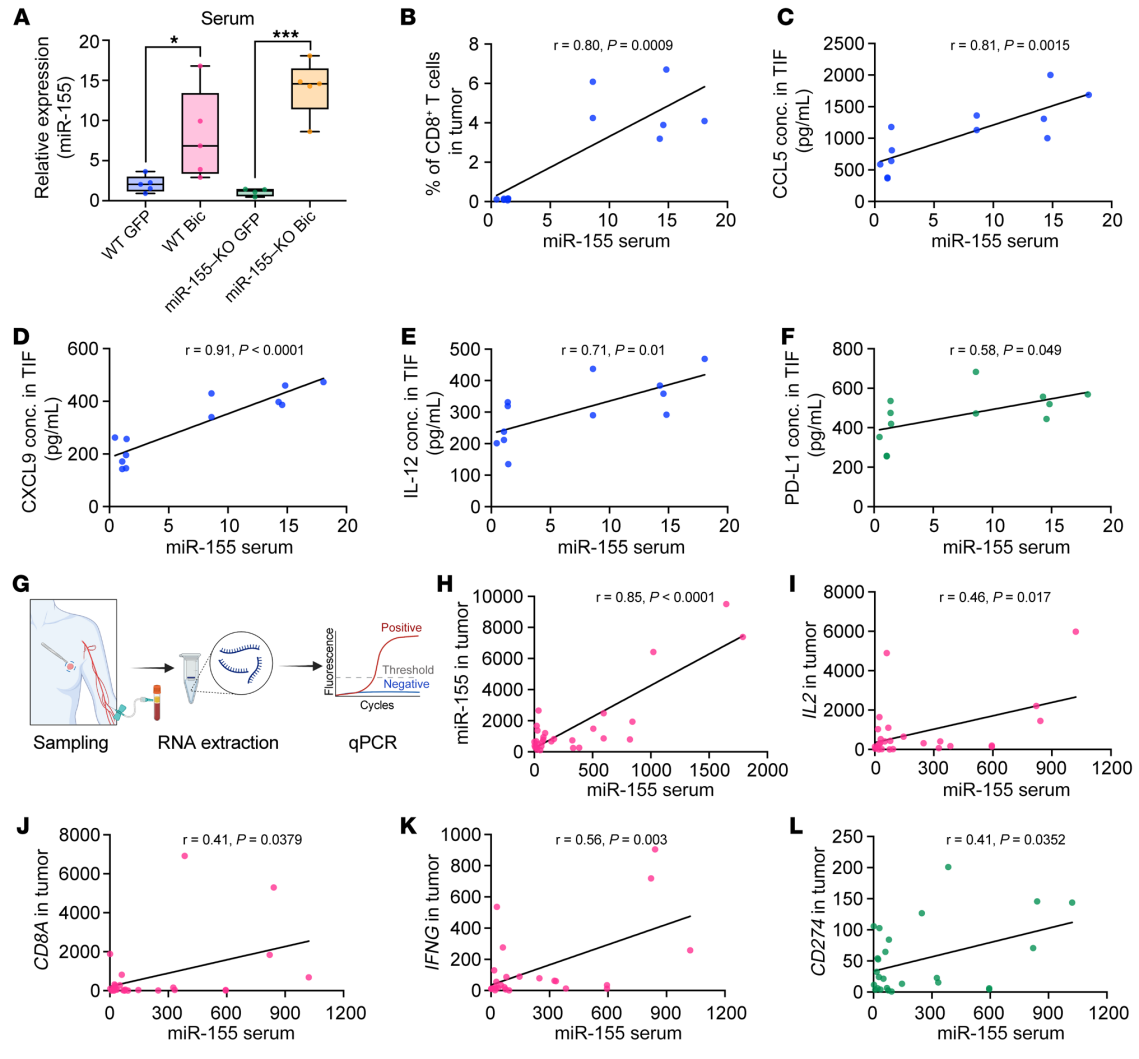
Circulating miR-155 mirrors antitumor immune status within breast tumors. (**A**) Relative miR-155 expression in serum collected from both WT and miR-155–KO mice carrying EO771-GFP or EO771-Bic tumors. *n* = 4 per group. Statistical significance was assessed using unpaired, 2-tailed Student’s *t* test, and all data are represented as mean ± SEM. *P < 0.05, ***P < 0.001. (**B**) Correlation between serum miR-155 levels measured by qPCR and the frequency of CD8^+^ T cells in tumors determined by flow cytometry. *n* = 12. (**C**–**F**) Correlation between serum miR-155 levels and CCL5 (**C**), CXCL9 (**D**), IL-12 (**E**), and soluble PD-L1 (**F**) concentrations in TIFs. *n* = 12. In a small cohort of human breast cancer samples, the expression of miR-155 and hallmark genes of T cell activation in serum, nontumor, and tumor tissues was determined by qPCR. (**G**) Schematic image showing the procedure of sampling from breast cancer patients. (**H**) Correlation between serum miR-155 levels and tumor tissue miR-155 expression. *n* = 29. (**I**–**L**) Correlations between serum miR-155 levels and mRNA levels of *IL2* (**I**), *CD8A* (**J**), *IFNG* (**K**), and *CD274* (**L**) in human breast cancer tumor tissues. *n* = 26. *P*and *r* values in **B**–**L** were calculated based on Pearson’s correlation analysis.

Tumor-derived nucleic acids, including miRNAs, have recently been proposed as diagnostic and prognostic biomarkers (33, 34). miR-155 was not only highly enriched in tumor-derived exosomes as mentioned above (Supplemental Figure 15B), but also detectable in the nonconcentrated cell culture media (Supplemental Figure 17, A–C). To test the feasibility of using circulating miR-155 levels as a prognostic biomarker, we then measured the miR-155 levels in serum of tumor-bearing mice. miR-155 was measured in the serum with significantly higher levels in EO771-Bic tumor–bearing mice than in EO771-GFP tumor–bearing mice (Figure 8A). Notably, the levels of serum miR-155 of WT and KO mice were comparable in mice with either EO771-GFP tumors or EO771-Bic tumors (Figure 8A). This suggests that breast cancer cells were the main source of serum miR-155 in these mice. A significant association was observed between serum miR-155 levels and the frequency of tumor-infiltrating CD8^^^+^^^ T cells in the tumors of miR-155–KO mice (Figure 8B). In addition, serum miR-155 levels were positively correlated with the protein levels of chemoattractant CCL5 and CXCL9 (Figure 8, C and D), immune-activating IL-12 (Figure 8E), and immunosuppressive PD-L1 (Figure 8F) in the TIFs of the miR-155–KO mice.

To explore the potential value of circulating miR-155 levels in evaluating the immune status of human breast tumors, we harvested matched sera and tumor tissues from a small cohort of patients with breast cancer (Figure 8G and Supplemental Table 1). Using qPCR to analyze miR-155 expression levels, we observed that, while serum miR-155 levels did not correlate with normal breast tissue miR-155 expression levels (Supplemental Figure 18), they faithfully reflected the miR-155 expression levels in breast tumor tissue (Figure 8H). Moreover, serum miR-155 levels were also positively correlated with the expression levels of hallmark antitumor immune activation genes *IL2*, *CD8A*, and *IFNG* (Figure 8, I–K). Notably, serum miR-155 abundance also mirrored the expression levels of the immunosuppressive molecule *CD274* (*PD-L1*) in tumor tissues (Figure 8L).

Taken together, these results indicate that circulating miR-155 can serve as a noninvasive biomarker in estimating the immune status of breast tumors and therefore may be of value in predicting their prognosis and response to ICB treatment.

## Discussion

miR-155 is a multifunctional molecule and plays intricate and sometimes contradictory roles in various cancers (7–9, 35–37). Recent findings of our group and others have revealed the pivotal role of immune cell–expressed miR-155 in antitumor immunity (10–15, 18, 19), while the functions of miR-155 expressed in breast cancer cells are more elusive. As the dynamic crosstalk between malignant and immune cells in the TME has a profound impact on tumor progression (38), in this study, we particularly sought to examine how breast cancer cell–derived miR-155 affects immune cell phenotype and functionality.

By analyzing human breast cancer data from multiple repositories in respect to miR-155 expression, we first demonstrated a correlation between higher miR-155 levels and favorable antitumor immune infiltrations in human breast tumors as well as better patient prognosis. To dissect a potential causative relationship, we investigated the direct role of cancer cell miR-155 in enhancing antitumor immunity using murine breast cancer models. It was found that overexpression of miR-155 in breast cancer cells substantially delayed tumor progression via increasing the recruitment of effector immune cells to the TME. Since this happened also in miR-155–deficient mice, which lack host miR-155 expression, the tumor-suppressive function of miR-155 overexpression in breast cancer cells is likely independent of miR-155 expression in immune cells. Furthermore, we found that miR-155 is secreted from breast cancer cells to the TME and circulation and thus circulating miR-155 may be utilized as a biomarker for evaluating the immune status of breast cancer patients.

There are some conflicting reports regarding the role of miR-155 in breast cancer development and progression. miR-155 expression levels in breast cancer have been shown to be associated with high-grade, advanced stage, metastases, and invasion (35, 39). However, a study on a large series of triple-negative breast cancers showed that high miR-155 levels decreased the efficiency of homologous recombination repair by targeting the recombinase RAD51 and thus were associated with better overall survival of patients (7). Another study reported that stable expression of miR-155 in 4T1 murine breast cancer cells significantly reduced the aggressiveness of tumor cell dissemination by preventing tumor cell epithelial-to-mesenchymal transition (EMT) in vivo (40). The seemingly contradicting conclusions reached in previous miR-155 studies may be attributed to variations in sample size, cancer types, animal models, and experimental design. Notably, we showed that even when miR-155 was expressed in breast cancer cells at a level 60-fold higher than baseline, it did not affect breast cancer cell proliferation and sensitivity to the chemotherapy drug doxorubicin.

We investigated the mechanism by which cancer cell miR-155 overexpression enhances antitumor immunity in breast tumors. Chemotactic cytokines and chemokines determine the migratory behavior of immune cells. Regarding the tumors, it has been well studied that CXCL9/10/11 (ligands for CXCR3) produced by macrophages and DCs, along with CCL5 (ligands for CCR5) secreted by tumor cells, are associated with T cell recruitment to the TME and a favorable response to chemotherapy and immunotherapy (41, 42). We observed that miR-155 dramatically upregulated CCL5 and CXCL9/10/11 expression in breast cancer cells. Accordingly, we also noted that conditioned medium from miR-155–overexpressing cells recruited more OVA-activated OT-1 T cells in vitro, suggesting that tumor cells with high miR-155 expression recruit more effective T cells to the tumor site and turn “cold” tumors “hot,” which may provide better targets for immunotherapy.

These findings prompted us to explore the intrinsic cellular signaling events regulated by miR-155. STATs are responsible for chemokine and cytokine production, which can be regulated by SOCS1. As SOCS1 is a direct miR-155 target, we hypothesized that miR-155 might increase CXCL9/10/11 expression by suppressing SOCS1 and thus regulating STAT activities. Indeed, we detected increased STAT1 expression and activation, but decreased p-STAT3 levels in miR-155–overexpressing cancer cells. Depending on the context, STAT1 and STAT3 play opposing roles and regulate one another via SOCS1. We confirmed the above regulatory mechanisms using human primary breast cancer cells overexpressing miR-155, miR-155–KO EO771 cells, as well as SOCS1-depleted EO771 cells. In addition, we also observed in our in vitro study that a STAT3 inhibitor increased CXCL9 expression in tumor cells, indicating the balance of p-STAT1/p-STAT3 was a determinant in regulating chemokine production in tumor cells.

Upon tumor-specific T cell activation, released IFNs trigger the inducible expression of immunosuppressive molecules by cancer cells or myeloid cells in the TME, thereby leading to T cell exhaustion and restricting the antitumor immune response, which is known as adaptive immune resistance (43). In the mice bearing miR-155–overexpressing tumors, along with the enhanced antitumor immune cell infiltration, a concomitant increase of immune-suppressive molecules, including PD-L1 and CTLA4, occurred. This would be expected to limit antitumor activity. These immune checkpoint genes were also found to be upregulated in miR-155^hi^ tumors from human breast cancer patients. We also discovered that miR-155 overexpression intrinsically increased PD-L1 expression in both human primary breast cancer cells and murine breast cancer cell lines, which may explain our in vivo findings showing the miR-155–overexpressing tumors are sensitive to ICB therapy. The establishment of immunological memory is an important aspect of durable antitumor responses against tumor relapse (29). In our study, we demonstrated that tumor-derived miR-155 was highly enriched in exosomes, which can significantly strengthen the antitumor immune response when applied to tumor-bearing mice i.v. We speculate that tumor-derived exosomal miR-155 may help the host to establish an augmented immune protective mechanism, as we observed that tumors with miR-155 overexpression elicited a stronger systemic immunological memory, resulting in rejection of rechallenged tumors after surgical removal of primary tumors.

Existing in free form or embedded in microvesicles, like the above-mentioned exosomes, miRNAs can be secreted into the circulation and exist in remarkably stable forms (34). An abundance of circulating miRNAs is in some cases associated with the initiation and progression of cancer and can be easily detected via basic molecular biology techniques (34, 44). Therefore, considerable effort has been devoted to identifying suitable circulating miRNAs as noninvasive biomarkers not only for early cancer diagnosis, but also as a predictor of prognosis and treatment response (33, 34). Our data showed that breast cancer cell–derived miR-155 can be released into peripheral blood and that serum miR-155 levels not only mirror the tumor miR-155 expression, but also reflect antitumor status in the TME. These data suggest the potential of utilization of circulating miR-155 levels in patients with breast cancer as a prognostic biomarker as well as a predictive marker for the efficacy of ICB therapies.

Also, importantly, because this study showed that the forced overexpression of miR-155 in breast cancer cells led to improved antitumor immunity accompanied by elevated expression levels of immune checkpoint molecules in breast tumors, we envision that nanotechnology or virus-based therapeutic strategies to increase miR-155 expression in breast tumors may enhance the efficacy of ICB therapies. A limitation of this study is that we based our conclusions on syngeneic breast cancer mouse models. In future studies, we will use humanized mice engrafted with human breast tumors to confirm our findings.

In conclusion, our study suggests that breast cancer cell–expressed miR-155 plays an antitumor role by enhancing antitumor immune responses, serum miR-155 levels can be used as a predictive biomarker for patient prognosis and response to immune therapy, and boosting miR-155 expression in breast tumors could be a promising therapeutic strategy, particularly when it is used in combination with ICBs.

## Methods

### Bioinformatic analysis.

RNA-Seq data, miRNA-Seq data, and clinical information for human breast cancer were retrieved from the Genomic Data Commons (GDC) portal (https://portal.gdc.cancer.gov/). After normalization using the limma R package, the expression data of miRNA-Seq and RNA-Seq were aligned to the clinical information for breast cancer patients (*n* = 995). miR-155^hi^ and miR-155^lo^ groups were separated based on the median value of miR-155 expression in tumors unless otherwise noted. The DEGs between these 2 groups were extracted using the DESeq R package, and the result was visualized in a volcano plot. The parameters set for differential expression analysis were FDR < 0.05 with |log_2_FC| > 1. Cox’s regression analysis was performed to assess the prognostic association of hsa–miR-155 expression, adjusted for age, sex, race, tumor stage, and tumor purity. Tumor purity was estimated by ESTIMATE (45), and the association analyses were performed on the SCISSOR platform (46). HR, 95% CI, and *P* value were calculated. The association of hsa–miR-155 expression with survival in each of 5 main intrinsic subtypes (luminal A, luminal B, HER2, basal-like, and normal-like), which were defined by PAM50 (47), was investigated. Patient survival data from the EGA repository (48) and the GEO data sets (49) were obtained from the KM plotter (50). For survival analysis, KM plot was generated by using the survival and survminer R packages and examined by using log-rank test.

### Pathway enrichment analysis.

A total of 12,888 genes with normalized values were analyzed for pathway and gene signature enrichment analysis. GO and KEGG pathway enrichment analyses were performed and visualized via GOplot and ggplot2 R packages. GSEA was performed using GSEA software (version 4.0.3) with the default settings and 1,000 gene set permutations. Gene sets used in these analyses were derived from the Molecular Signature Database (MSigDB) (51). Multi-GSEA plots were generated using the ggplot2 R package.

### Immune cell infiltration with CIBERSORTx.

CIBERSORTx was applied to estimate the immune cell composition of breast cancer tumor tissues based on a validated leukocyte gene-signature matrix and default settings. The normalized gene expression profile of TCGA data was input into CIBERSORTx for analysis based on a deconvolution algorithm with 100 permutations and S-batch correction to remove variances between different sequence platforms. To control the accuracy of the deconvolution algorithm, data with a *P* value of less than 0.05 were screened for the following analysis.

### Patients and specimens.

Matched tumor samples for serum and tumor miR-155 expression analysis were obtained from patients with pathologically confirmed breast cancer at Nanjing Drum Tower Hospital, the Affiliated Hospital of Nanjing University Medical School. None of the patients received anticancer therapy prior to sampling. Paired serum, nontumor (taken at least 3 cm distal to the tumor site), and tumor tissues from 29 breast cancer patients who underwent a tumor resection (Supplemental Table 1) were used to extract RNA. The TNM staging was classified according to the eighth edition of the American Joint Committee on Cancer (AJCC) (52). Triple negative breast cancer samples for primary cancer culture were obtained from the Cancer Institute of Prisma Health.

### Mice.

All mice used for this study were 6- to 8-week-old females, including C57BL/6 WT, miR-155–KO, and OT-1 mice, all of which were obtained from Jackson Laboratories. Mice were maintained in pathogen-free conditions at the University of South Carolina according to NIH guidelines.

### Cell culture and tumor conditioned medium collection.

Breast cancer cell lines EO771 (ATCC, CRL-3461), 4T1 (ATCC, CRL-2539), and AT-3 (Sigma-Aldrich, SCC178) and melanoma cell line B16-F10 (ATCC, CRL-6475) were expanded in high-glucose DMEM (Sigma-Aldrich, D6429) supplemented with 10% FBS (Gibco, Thermo Fisher Scientific; A4766801), 100 U/mL penicillin, and 100 μg/mL streptomycin (Gibco, Thermo Fisher Scientific; 15140148). All cells were maintained in a humidified, 5% CO_2_ incubator at 37°C.

For in vitro CXCL9 level determination, tumor cells were treated with Stattic (Sigma-Aldrich, S7947), as indicated, and then cells were trypsinized and harvested for flow cytometry. For chemo-sensitivity assay, tumor cells were treated with doxorubicin for 24 hours and cell viability was determined by MTT assay (Sigma-Aldrich, CT02) according to the manufacturer’s instructions.

Tumor-conditioned medium was prepared by plating 6 × 10^^^6^^^ tumor cells in 10 cm dishes. The medium was changed to serum-free DMEM for another 48 hours when cells were 80% confluent. The supernatant harvested was filtered by 0.45 μm strainer and stocked at –80°C as tumor-conditioned medium.

### miR-155–overexpressing tumor cell line establishment.

Details of lentiviral vector construction and lentiviral transduction have been reported previously (14, 15). Forty-eight hours after transduction, GFP^+^ cells were sorted for the subsequent experiments.

### miR-155 KO cell generation by CRISPR-Cas9.

For the generation of lentiviral-based miR-155 KO via CRISPR/Cas9, EO771 cells were transfected with empty lentiCRIPSR or lentiCRISPR containing dual-gRNA (5′-GTTGCATATCCCTTATCCTC-3′ and 5′-GACATCTACGTTCATCCAGC-3′) targeting miR-155. After limiting dilution, a single clone was selected out in the presence of puromycin (2 μg/mL) for 3 weeks. qPCR was performed to validate the successful miR-155 KO in EO771 cells before using for downstream experiments.

### Human primary breast cancer cell isolation and culture.

Triple-negative human breast cancer cells were isolated and cultured as previously reported (53). Briefly, tumor tissue was mechanically cut into small pieces (<1 mm^3^) using scalpels and enzymatically dissociated in a mixture of collagenase/hyaluronidase (STEMCELL Technologies, 07912) for 16 hours at 37°C, followed by further digestion with trypsin (0.25%) for 2 minutes and then 5 units/mL of dispase (STEMCELL Technologies, 07913) and 0.05 mg/mL of DNase I (STEMCELL Technologies, 07900) for 1 minute. After centrifugation at 150*g* for 5 minutes, cells were seeded at a density of 1 × 10^5^/well onto Geltrex-coated (23 μg of protein per 1 cm^2^; Thermo Fisher Scientific, A1413202) 6-well plates in human complete EpiCult-B medium (STEMCELL Technologies, 05602, 05630, and 07925). Cells were either further cultured or passaged using trypsin-EDTA (0.25%) for downstream usage.

### Tumor models.

Mouse orthotopic breast cancer models were established as previously described with a minor modification (12, 14). Briefly, 2 × 10^5^ EO771 cells suspended in 10 μL PBS were implanted into the fourth pair of mammary fat pads of mice. To establish s.c. melanoma in mice, 1 × 10^6^ B16-F10 cells in 10 μL of PBS were implanted into the rear flanks of mice. The tumor size was monitored by caliper on indicated days. Tumor volume was calculated according to the following formula: tumor volume ≈ (short axis)^2^ × (long axis)/2. To determine tumor sensitivity to immunotherapy, anti-mouse PD-L1 mAbs (100 μg/mouse, BioXcell, BP0101) were applied to mice with established EO771-GFP or EO771-Bic tumors i.v. on day 12, day 15, and day 18 after tumor inoculation; IgG2b was used as isotype control (BioXcell, BP0090). Tumor volume was monitored. Tumor sensitivity to anti–PD-L1 treatment was determined using the following formula:

 (Equation 1)
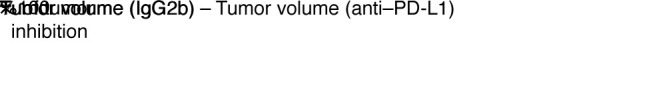


At the experimental end point, mice were sacrificed. Tumors and tumor-draining lymph nodes were removed, weighed, and processed for subsequent experiments.

### Cell isolation and interstitial fluid collection.

Cells from mouse spleens were isolated by mechanical disruption. Tissue-infiltrating leukocytes were obtained as described previously (14, 54). In short, fresh resected tumor specimens were minced and enzymatically digested in completed RPMI 1640 medium (Sigma-Aldrich, R8758) supplemented with 0.3 mg/mL of collagenase, type 4 (Worthington, LS004189), 200 U/mL of DNase I (Worthington, LS006334), and 1 U/mL of hyaluronidase (Sigma-Aldrich, H3506) for 1 hour at 37°C. Cells were thoroughly rinsed with ice-cold PBS; then erythrocytes were lysed using red blood cell lysing buffer (Sigma-Aldrich, R7757) per the manufacturer’s instructions. Dissociated cells were passed through a 70 μm cell strainer and resuspended in medium supplemented with 1% FBS for flow cytometry analysis.

Tumor-infiltrating leukocytes were isolated using the EasySep Mouse PE Positive Selection Kit (STEMCELL Technologies,17696) following the manufacturer’s instructions. Splenic T cells were isolated using the EasySep Mouse T Cell Isolation Kit (STEMCELL Technologies,19851) following the manufacturer’s instructions. In all sorted samples, a purity of greater than 95% was achieved as determined by flow cytometry.

To collect TIFs, tumors were freshly harvested and cut into small pieces. Samples were then vortexed in serum-free DMEM (0.5 g tissue/mL medium) for 30 seconds to dissolve the interstitial fluid and centrifuged at 450*g* for 5 minutes. The resulting supernatant was collected and filtered through a 0.22 μm filter to remove any debris. The obtained liquid was referred to as TIFs.

### Flow cytometry.

Flow cytometry was performed as previously described (14, 54). For surface staining, the in vitro–cultured and tissue-infiltrating cells were stained with fluorochrome-conjugated Abs for 30 minutes, then washed and analyzed via flow cytometry. For intracellular staining, cells were stimulated with or without Cell Activation Cocktail (BioLegend, 423304), depending on the experiment’s needs, followed by fixation and permeabilization treatment via manufacturer protocols. Samples were then incubated with fluorochrome-conjugated Abs for 30 minutes, washed, and resuspended in wash buffer. To measure cell-proliferation capacity, we performed a BrdU incorporation assay. For in vitro labeling, 1 μM of BrdU was applied 30 minutes prior to harvesting. For in vivo labeling of mouse cells, 1 mg BrdU in 200 μL of PBS was injected i.p. 24 hours prior to harvesting. All samples were then processed according to the manufacturer’s directions (BD Biosciences, 559619). To evaluate apoptosis, cells were incubated with 5 μg/mL of propidium iodide (PI) (BioLegend, 421301) for 15 minutes before analysis. Data were acquired and read on a BD FACS Aria II Flow Cytometer and analyzed using FlowJo software, version 10.8.0 (BD Biosciences). Details of the fluorochrome-conjugated Abs that were used in this study are listed in Supplemental Table 4.

### qPCR for mRNA and miR-155 expression.

Tissues/cells were lysed in 700 μL QIAzol lysis reagent (QIAzol), and tissue samples were homogenized. RNA was extracted using QIAGEN miRNeasy Mini Kits (217084) to allow for the collection of microRNAs and mRNAs. cDNA was then synthesized with 1 μg RNA using miScript II RT Kits (QIAGEN, 218161). miR-155 expression was measured by a Bio-Rad CFX96 thermocycler using miScript SYBR Green PCR Kits (QIAGEN, 1046470) according to the manufacturer’s instructions. QIAGEN miScript primers were purchased for mmu–miR-155 (mouse, MS00001701), Hsa-miR-155 (human, MS00003605), and RNU6 (MS00033740). For normalization, miR-155 expression was presented relative to RNU6 expression.

For mRNA expression detection, qPCR was performed using iQ SYBR Green Supermix (Bio-Rad, 1708880). All primers used for qPCR analysis of genes were synthesized by Integrated DNA Technologies. The primer sequences are listed in Supplemental Table 5. The relative amount of target mRNA was determined using the Ct method by normalizing target mRNA Ct values to those of 18s rRNA.

### LEGENDplex assay.

To investigate the cytokine/chemokine profile in the secretomes of the EO771-GFP and EO771-Bic cells, we performed a multiplex proinflammatory chemokine panel assay (BioLegend, 740451) according to the manufacturer’s instructions. The concentration of chemokines was quantified using the LEGENDplex Data Analysis Software Suite, version 2022-07-15.

### ELISA.

The concentrations of IFN-γ (BioLegend, 430804), TNF-α (BioLegend, 430904), CCL5 (R&D, DY478-05), CXCL9 (R&D, DY492-05), and soluble PD-L1 (R&D, DY1019-05) in tumor-conditioned media or TIFs were determined using ELISA kits according to the manufacturers’ instructions.

### In vitro T cell activation and migration.

T cell activation and migration assays were performed following the protocol by Albert et al. (23). Briefly, splenocytes from OT-1 transgenic mice were stimulated with 1 nM TCR-specific peptide ovalbumin 257–264 ( Sigma-Aldrich, S7951) for 1 hour. Seven days after culture in complete RPMI 1640 medium with 50 U/mL of recombinant IL-2 (R&D, 402-ML-020/CF), CD8^+^ T cells with high CXCR3 expression were used for T cell migration assay. To inhibit receptor binding of CXCR3, anti-mouse CXCR3 mAbs (10 μg/mL, BioXcell, BE0249) were applied to treat activated OT-1 cells for 1 hour at 37°C; IgG2b-pretreated (10 μg/mL, BioXcell, BP0090) cells were used as isotype control. Then, 0.1 × 10^6^ activated T cells were placed into 5 μm pore size polystyrene Transwell inserts (Corning, 3421) in serum-free RPMI 1640 and allowed to migrate for 1.5 hours at 37°C toward EO771-GFP/Bic cell conditioned medium. Cells that had migrated to the receipt chamber were collected and counted using Precision Counting Beads (BioLegend, 424902) by flow cytometry.

### TUNEL assay.

Apoptosis in tumor sections was determined using the In Situ Cell Death Detection Kit according to the manufacturer’s instructions (Roche, C755B40). Dead cells were quantified by counting the number of TUNEL^+^ cells in 10 fields for each section.

### Western blotting.

In vitro–cultured cancer cells from independent dishes were dissolved in RIPA cell lysis buffer (Thermo Fisher, 89901) supplemented with a protease inhibitor (Sigma-Aldrich, P8340) and a phosphatase inhibitor (Sigma-Aldrich, P00441). The protein concentrations were determined using a Rapid Gold BCA Protein Assay Kit (Thermo Fisher, A53227). In each group, equal amounts of protein were separated by SDS/PAGE and transferred to a 0.22 or 0.45 μM nitrocellulose (50) membrane by electroblotting. Samples were loaded either on the same gel or separate gels. For antigens that were detected on different blots, separated loading controls were applied. The indicated Abs (Supplemental Table 6) were applied, and the protein bands were determined using ECL Plus Reagent (Thermo Fisher, 32132). Western blotting bands were quantified using ImageJ software (NIH) relative to internal control (β-actin) expression.

### SOCS1 knockdown by siRNA.

To silent SOCS1 expression, EO771 cells were transfected with 10 nM of siRNA target mouse SOCS1 (OriGene, SR426031) using Lipofectamine RNAiMAX Reagent (Invitrogen, 13778) according to the manufacturer’s instructions. Forty-eight hours after transfection, cells were harvested for downstream analysis.

### Exosome purification and in vivo administration.

For exosome purification from EO771-GFP/Bic cell conditioned media, extracellular vesicles were purified by a standard differential centrifugation protocol. In brief, culture supernatants were centrifuged at 2,000*g* for 10 minutes to remove cell debris and dead cells. Microvesicles were next pelleted by centrifugation at 10,000*g* for 30 minutes. Supernatants were then centrifuged at 160,000*g* for 90 minutes at 4°C. The pelleted exosomes were suspended in PBS. The size distribution of isolated exosomes was measured using nanoparticle tracking analysis. The purified exosomes were quantified by determining protein concentrations using a Rapid Gold BCA Protein Assay Kit (Thermo Fisher, A53227).

For in vivo study, GFP or Bic exosomes with 50 μg of protein equivalent in 100 μL of PBS were injected via retroorbital venous plexus on EO771 tumor–bearing mice. Three days later, tumors and tumor-draining lymph nodes were removed for immune profile analysis.

### Statistics.

Data are shown as mean ± SEM whenever the mean is the primary value representative of a sample group’s behavior. Two-group comparison was accomplished using a 2-tailed Student’s *t* test or Wilcoxon’s rank sum test as indicated. One-way ANOVA followed by Tukey’s post hoc test was used for multiple comparisons. Two-way ANOVA with Tukey’s post hoc test was used to analyze tumor growth data. Cumulative survival time was estimated using the KM method. The survival association analysis was performed using the Cox’s proportional hazards model. Comparisons were performed using GraphPad Prism 9 (Graphpad Software Inc.) or R. *P* ≤ 0.05 was considered statistically significant for all tests.

### Study approval.

All animal experiments were approved by the IACUC at the University of South Carolina. For experiments using human breast cancer specimens, all samples were anonymously coded as stipulated by the Declaration of Helsinki. Written, informed consent was obtained from the patients prior to inclusion in the study. The use of human subjects for this study was approved by the IRB of Nanjing Drum Tower Hospital and Prisma Health.

## Author contributions

JW and DF designed the experiments. JW, QW, XW, KL, and YW performed experiments and analyzed and interpreted data. YG, YS, and YY provided clinical samples and collected data of human specimens. JW and GC performed bioinformatics analysis. ML, JAK, EAM, and YY helped in data interpretation. JW, KL, and DF wrote the manuscript, and all authors contributed to editing of the manuscript.

## Supplementary Material

Supplemental data

## Version 1 08/04/2022

In-Press Preview

## Version 2 10/03/2022

Electronic publication
