# Supplementary material for: Breast cancer cell–derived microRNA-155 suppresses tumor progression via enhancing immune cell recruitment and antitumor function
Source: J Clin Invest. 2022 Oct 3;132(19):e157248. doi: 10.1172/JCI157248 (PMC9525116; doi:10.1172/JCI157248)
Supplement: Supplemental data [file jci-132-157248-s041.pdf]

Breast cancer cell-derived microRNA-155 suppresses tumor progression via enhancing immune cell recruitment and function

Contents

Supplementary figures 2

Supplementary tables 23

Supplementary Figures

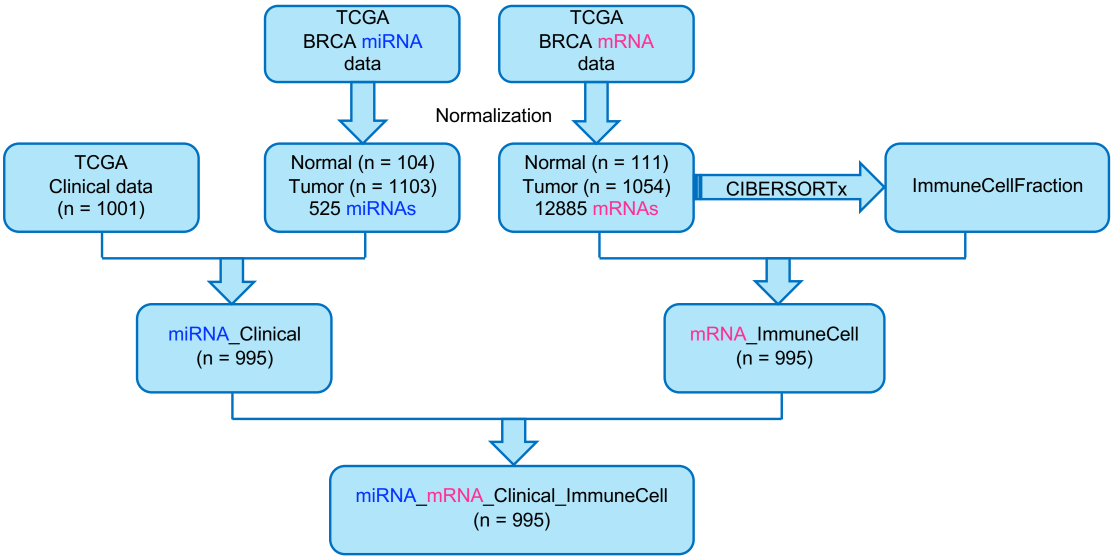

Supplementary Figure 1. **Flow chart of bioinformatic analysis.**
Abbreviations: TCGA: The Cancer Genome Atlas project; BRCA: breast cancer.

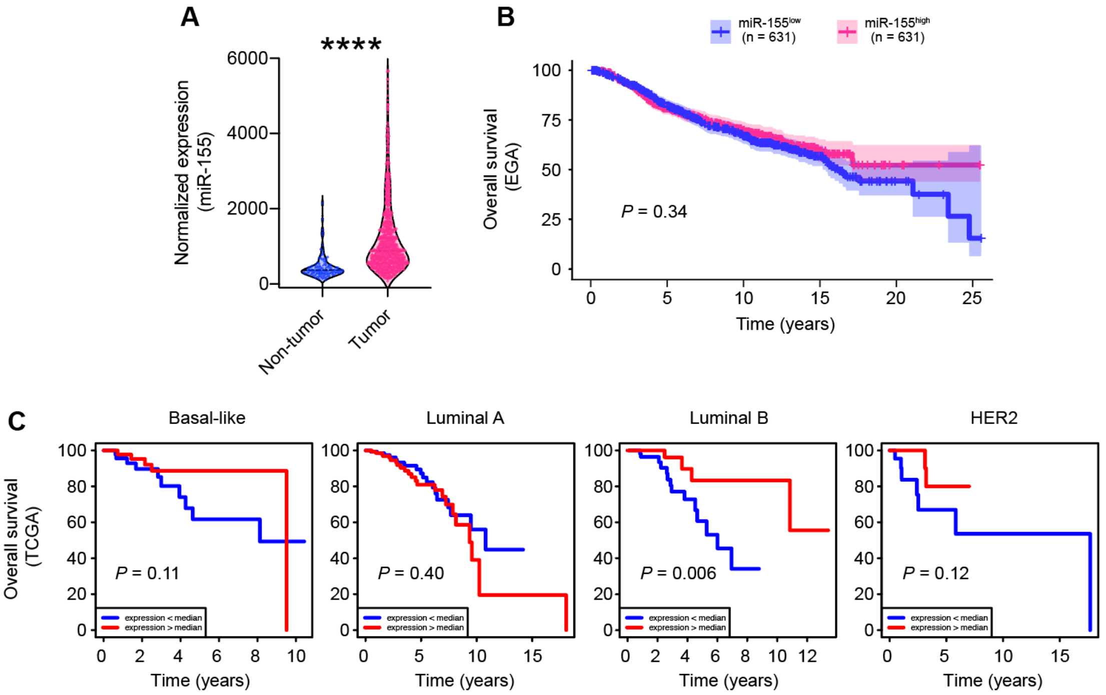

Supplementary Figure 2. **miR-155 is highly expressed in breast tumors and positively associated with better patient outcome.** (**A**) Normalized miR-155 expression in human breast tumors (n = 995) and adjacent non-tumor tissues (n = 99) (TCGA data). Statistical significance was assessed using unpaired, two-tailed, Student's *t*-test and all data points are presented as mean ± SEM. ****$P < 0.0001$. (**B**) Overall survival of BRCA patients of EGA cohort. Patients were divided into 2 groups according to the median value of normalized miR-155 level in tumors. (**C**) Overall survival of BRCA patients of TCGA cohort with different molecular classifications and miR-155 expression levels based on the median value; miR-155^{low} (blue), miR-155^{high} (red). The curve comparison with the log-rank (Mantel-Cox) test revealed statistically significant differences as shown on graphs.

TCGA, The Cancer Genome Atlas; BRCA, breast cancer; EGA, European Genome-Phenome Archive.

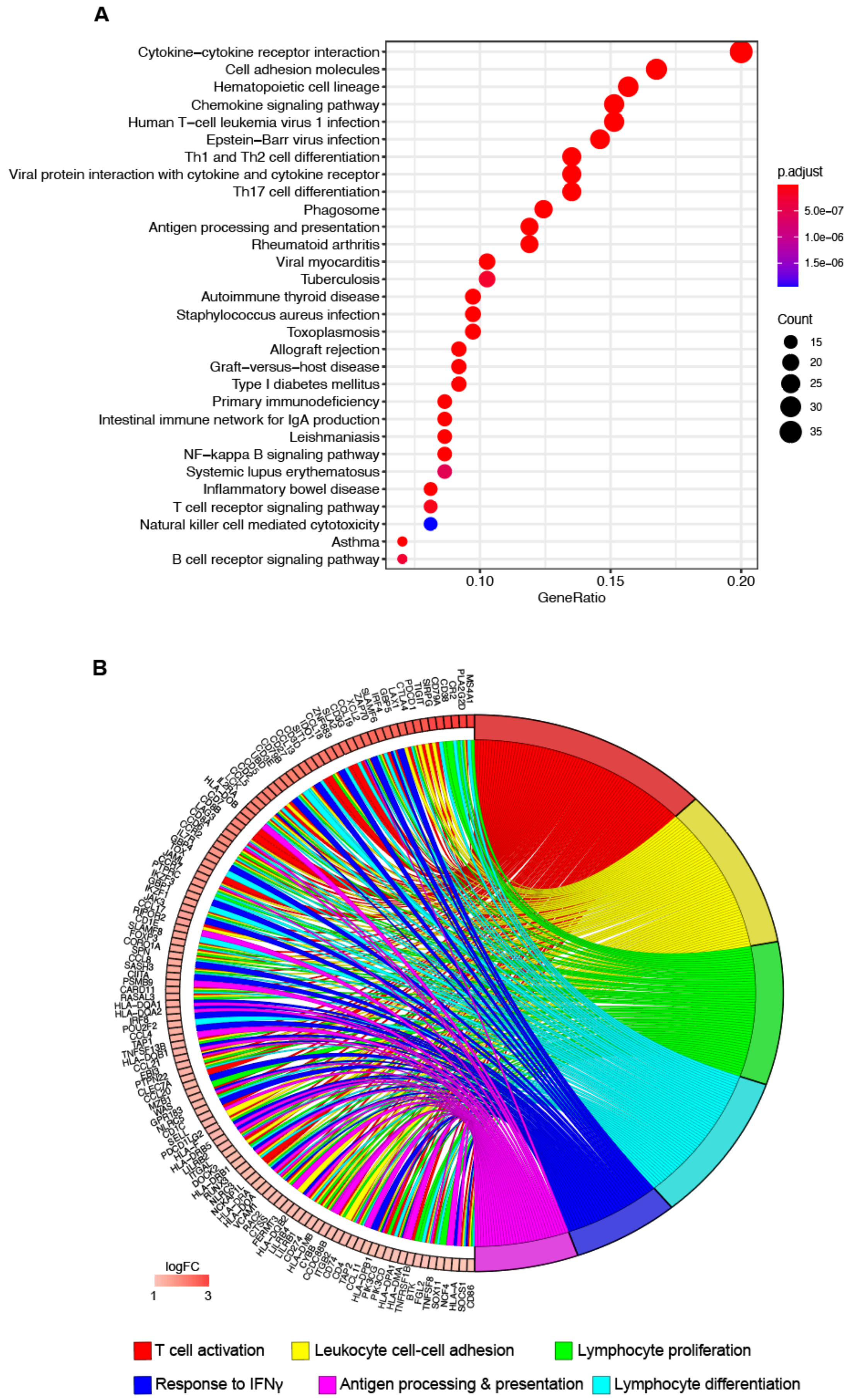
A
Cytokine−cytokine receptor interaction
Cell adhesion molecules
Hematopoietic cell lineage
Chemokine signaling pathway
Human T−cell leukemia virus 1 infection
Epstein−Barr virus infection
Th1 and Th2 cell differentiation
Viral protein interaction with cytokine and cytokine receptor
Th17 cell differentiation
Phagosome
Antigen processing and presentation
Rheumatoid arthritis
Viral myocarditis
Tuberculosis
Autoimmune thyroid disease
Staphylococcus aureus infection
Toxoplasmosis
Allograft rejection
Graft−versus−host disease
Type I diabetes mellitus
Primary immunodeficiency
Intestinal immune network for IgA production
Leishmaniasis
NF−kappa B signaling pathway
Systemic lupus erythematosus
Inflammatory bowel disease
T cell receptor signaling pathway
Natural killer cell mediated cytotoxicity
Asthma
B cell receptor signaling pathway
0.10
0.15
0.20
GeneRatio
p.adjust
5.0e−07
1.0e−06
1.5e−06
Count
15
20
25
30
35
B
logFC
1
3
T cell activation
Leukocyte cell-cell adhesion
Lymphocyte proliferation
Response to IFNγ
Antigen processing & presentation
Lymphocyte differentiation

Supplementary Figure 3. High miR-155 level in BRCA tumor defines an anti-tumor immune profile. Bubble plot of the KEGG enrichment analysis (**A**) and circos plot of the Gene Ontology enrichment analysis (**B**) using the differentially expressed genes between miR-155^{high} (n = 497) and miR-155^{low} (n = 498) tumor tissues.

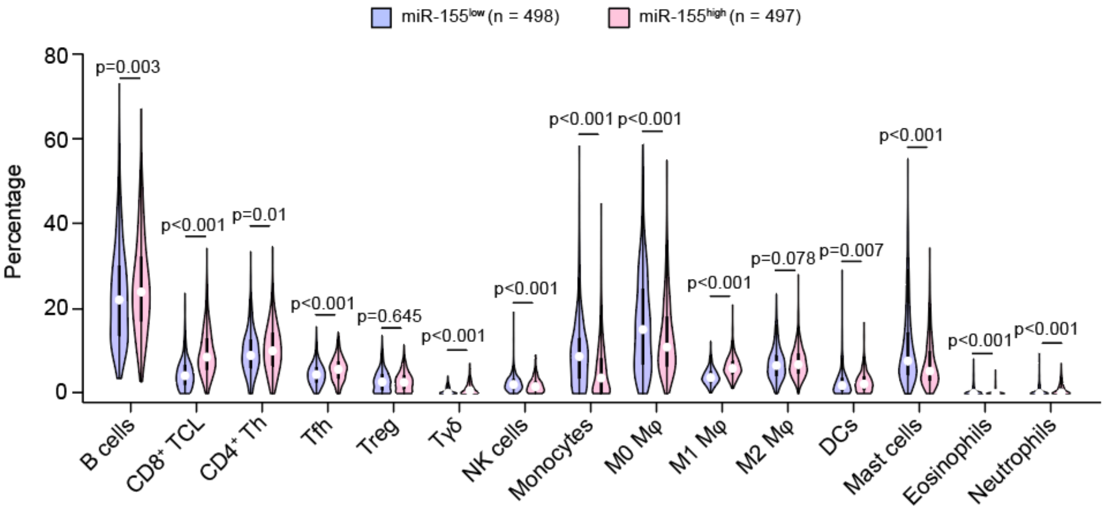

Supplementary Figure 4. miR-155 levels are positively associated with tumor infiltrating immune cells in BRCA tumors. Predicted fractions of 15 types of immune cells in tumors with different miR-155 expression via CIBERSORTx, Wilcoxon rank sum test was carried out to compare the frequencies of immune cells between miR-155^{high} and miR-155^{low} BRCA tumors, *P* values are shown.

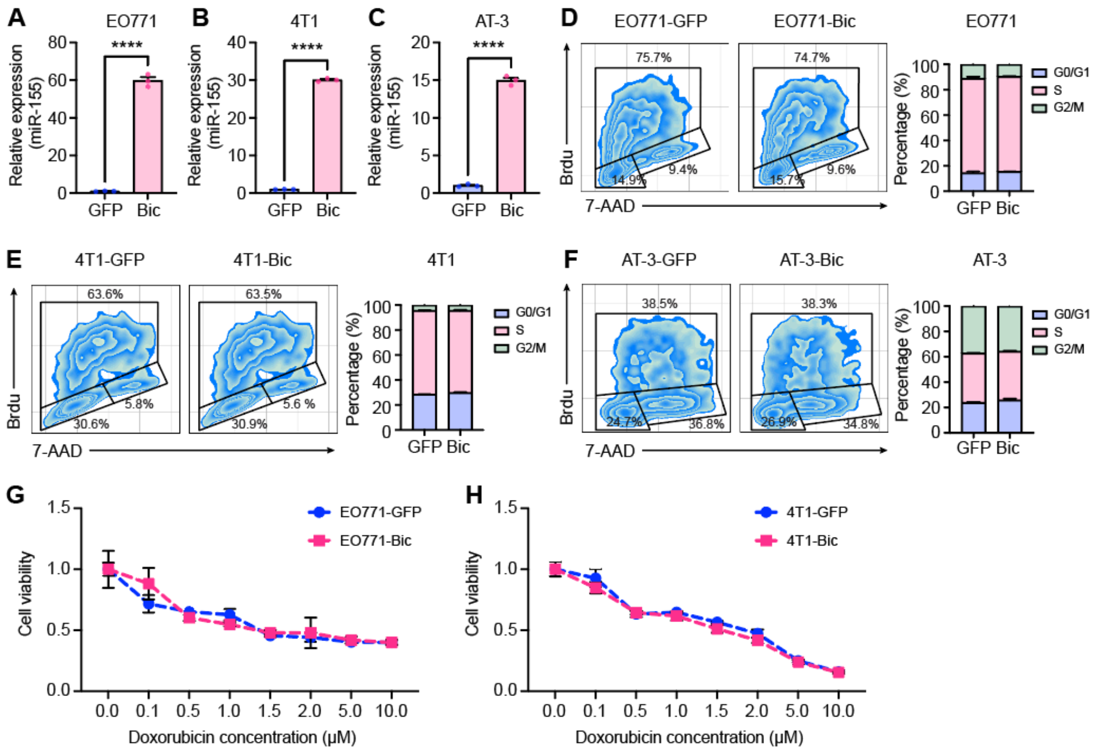

Supplementary Figure 5. Overexpression of miR-155 did not affect breast cancer cell proliferation and responsiveness to doxorubicin. Confirmed miR-155 expression in Bic lentivirus transduced EO771 (**A**), 4T1 (**B**) and AT-3 (**C**) breast cancer cells, cells transduced with GFP lentivirus were used as controls. In vitro BrdU incorporation assay of EO771-GFP and EO771-Bic cells (**D**) 4T1-GFP and 4T1-Bic cells (**E**) and AT-3-GFP and AT-3-Bic cells (**F**); representative zebra flow plots (left) showing BrdU^{+} cells and populations at different cell cycles. EO771-GFP and EO771-Bic (**G**) and 4T1-GFP and 4T1-Bic cells (**H**) were treated by various concentrations of doxorubicin for 24 hours; cell viabilities were determined by MTT assay. n = 3 for all experiments. Statistical significance was assessed using unpaired, two-tailed, Student's *t*-test and all data points are presented as mean ± SEM. ****$P < 0.0001$.

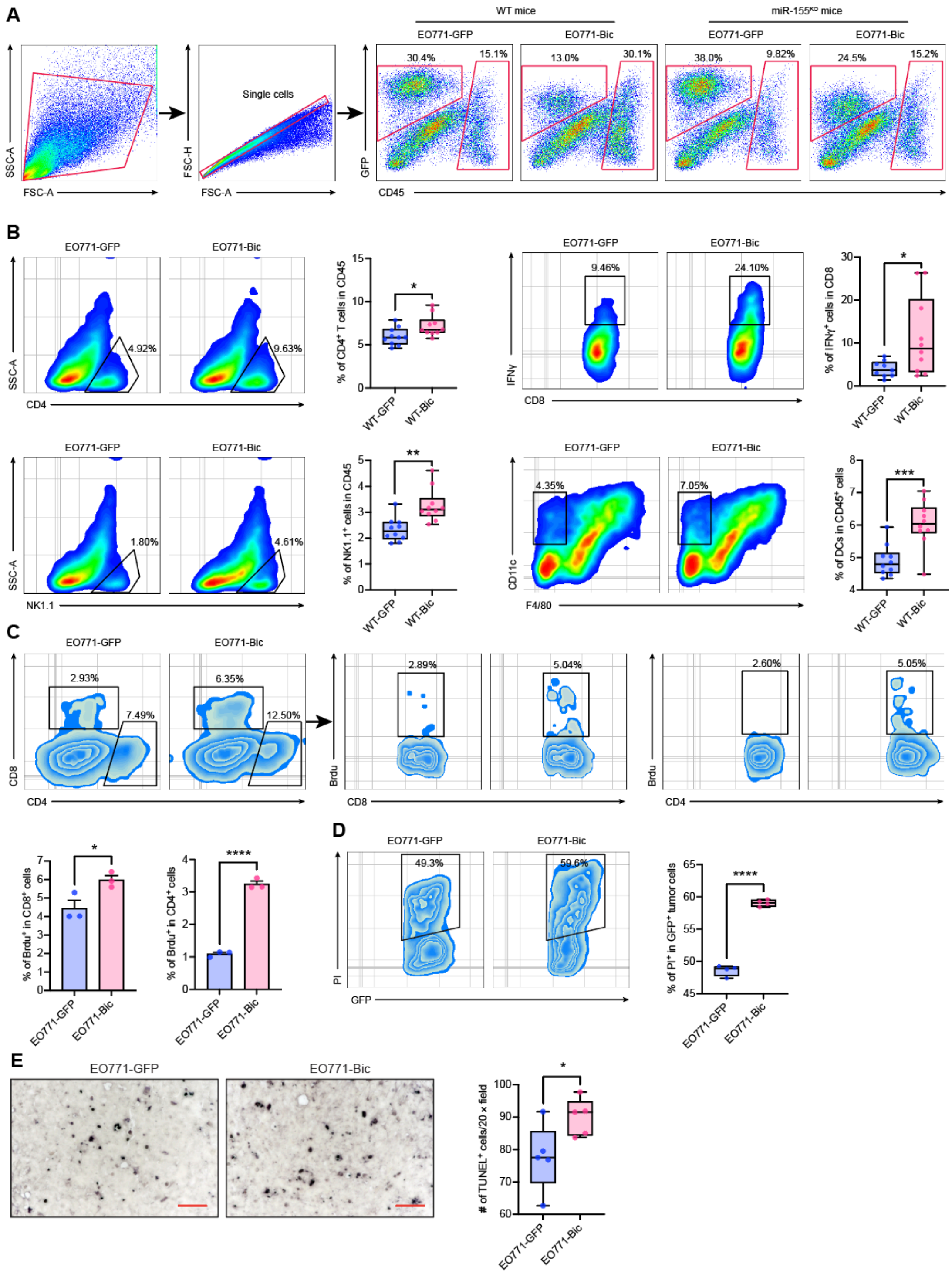

Supplementary Figure 6. **miR-155 overexpression in tumors augments anti-tumor immunity.** (**A**) A representative gating strategy illustrating immune cells ($CD45^+$) and tumor cells (GFP^+) being sub-gated to subsequent analysis. (**B**) The representative flow images and quantified statistics showing the percentages of $CD4^+$ T cells, IFNγ producing $CD8^+$ T cells, NK cells and DCs gated on $CD45^+$ leukocytes. n = 10 per group. (**C**) Gating strategy illustrating $Brdu^+$ T cells retrieved from tumor tissues. Bar graphs showing the frequencies of proliferating $CD4^+$/$CD8^+$ T cells. n = 3 per group. (**D**) PI^+ apoptotic tumor cells in EO771-GFP or EO771-Bic tumor tissues. n = 4 per group. (**E**) TUNEL staining of paraffin embedded tumor slides showing apoptotic cells in situ. n = 5 per group. Scale bar: 100 μM. Statistical significance in

this figure was assessed using unpaired, two-tailed, Student's *t*-test and all data points are presented as mean ± SEM. *$P < 0.05$, **$P < 0.01$, ***$P < 0.001$, ****$P < 0.0001$.

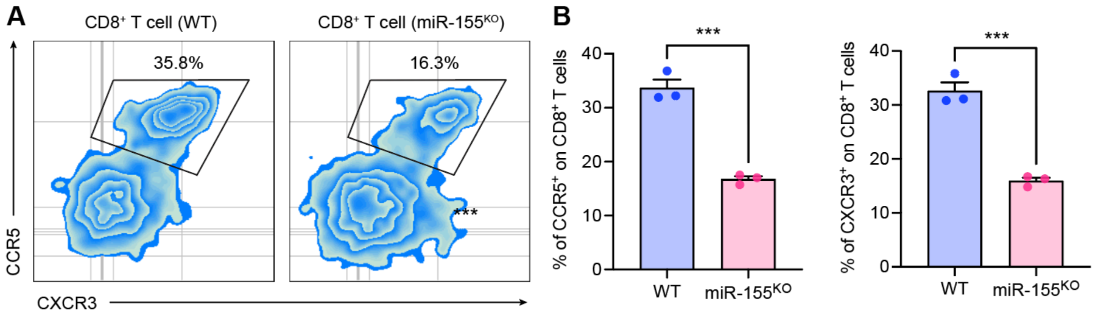

Supplementary Figure 7. miR-155 deficiency impairs CCR5/CXCR3 expression on T cells. T cells purified from splenocytes of WT or miR-155^{KO} mice were stimulated with anti-CD3 (2 μg/ml) and anti-CD28 (2 μg/ml) antibodies for 72 h. The cells were then collected, stained with fluorescence conjugated antibodies, the expression of CCR5 and CXCR3 on CD8^{+} T cells was analyzed by flow cytometry. The representative contour images (**A**), and quantified frequencies of CCR5 and CXCR3 positive CD8^{+} T cells (**B**) are shown. n = 3 per group. Statistical significance was assessed using unpaired, two-tailed, Student's *t*-test and all data points are presented as mean ± SEM. ***P < 0.001.

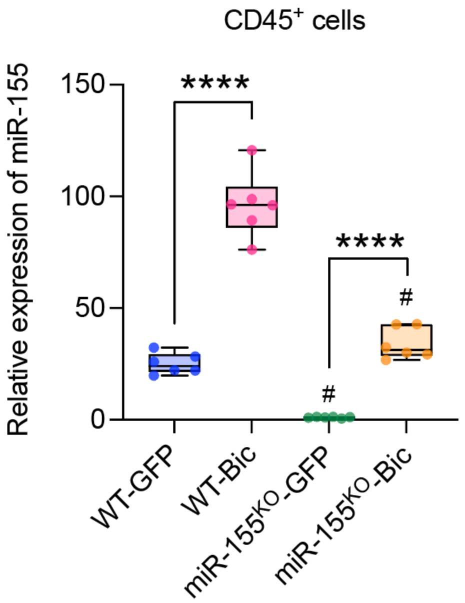

Supplementary Figure 8. **miR-155 expression in CD45$^+$ cells isolated from tumor tissue.** Tumor infiltrating CD45$^+$ cells were sorted using magnetic beads, miR-155 expression levels were determined by qPCR. n = 5 per group. Statistical significance was assessed using one-way ANOVA followed by Tukey's post-hoc test. all data points are presented as mean ± SEM. ****P < 0.0001. # denotes P < 0.05 compared to WT counterparts

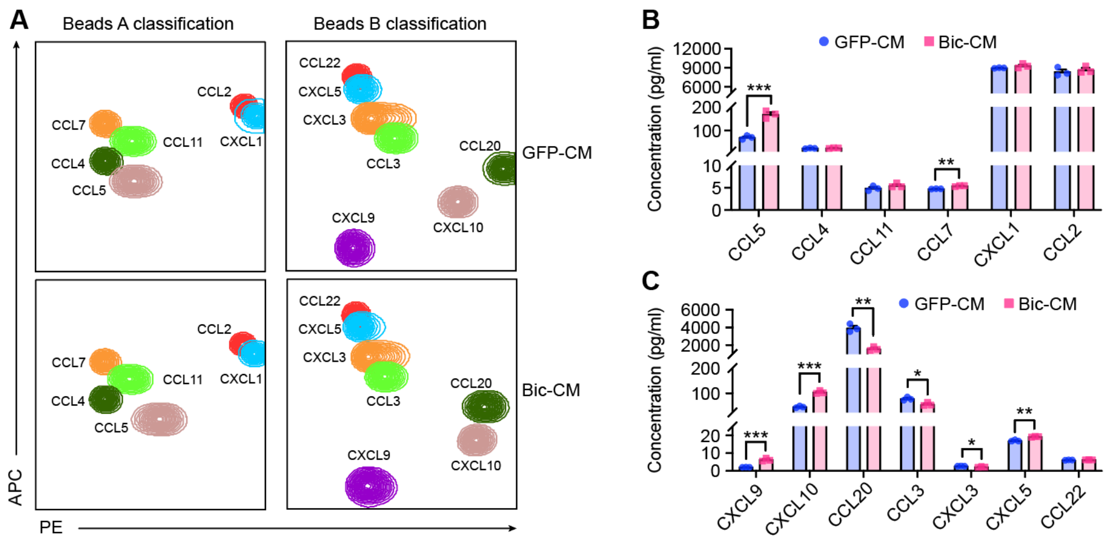

Supplementary Figure 9. **Proinflammatory chemokine panel in EO771-GFP and EO771-Bic conditioned media.** (**A**) Representative contour images of flow cytometry showing the distribution (APC channel) and fluorescence intensities (PE channel) of 13 types of chemokines. Quantified chemokines under beads A classification (**B**) and beads B classification (**C**), respectively. n = 3 per group. Statistical significance in this figure was assessed using the unpaired, two-tailed, Student's *t*-test and all data points are presented as mean ± SEM. $^*P < 0.05$, $^{**}P < 0.01$, $^{***}P < 0.001$. CM: conditioned medium.

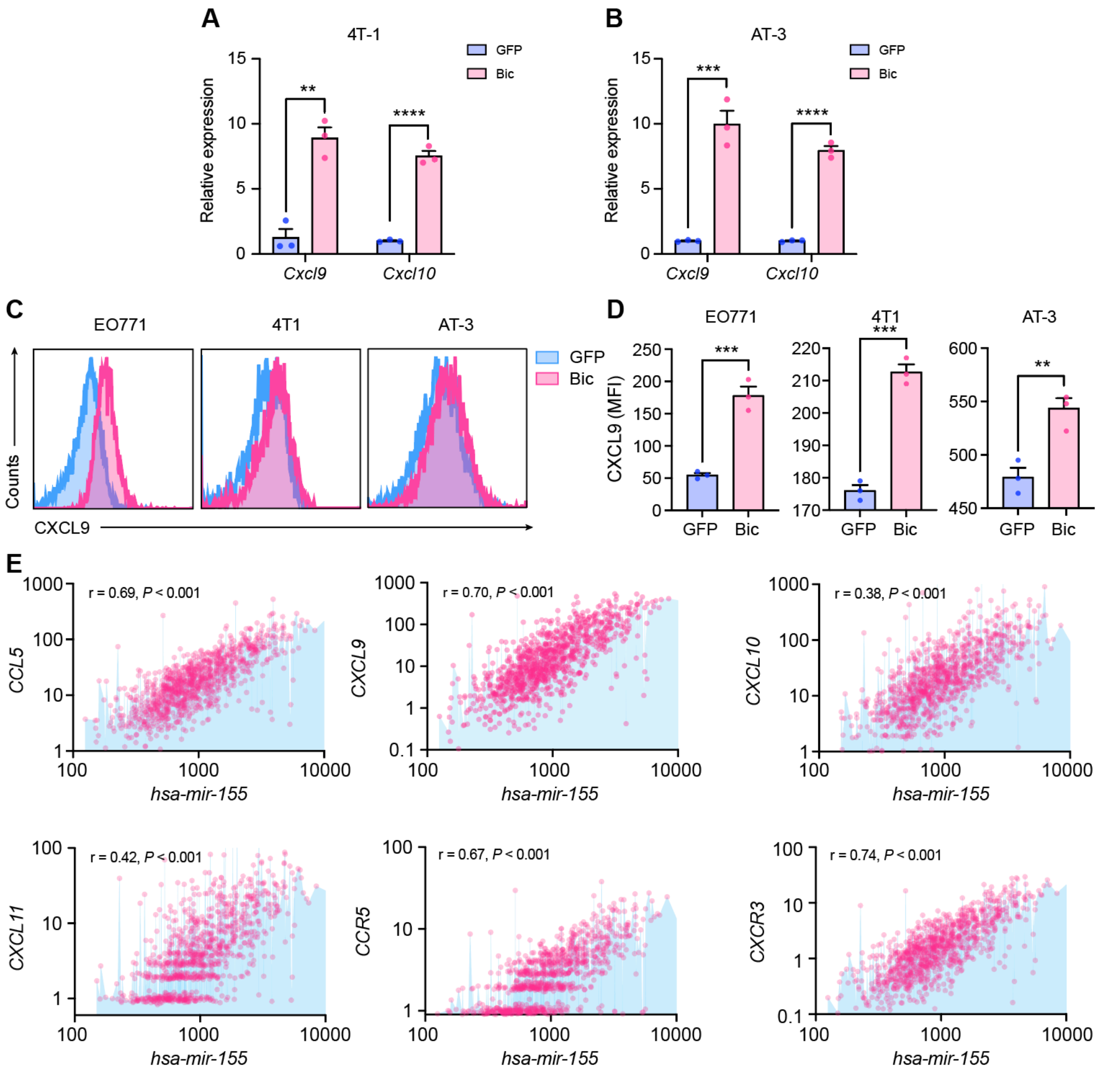

Supplementary Figure 10. The correlations of miR-155 expression and T cell recruitment genes. (**A**) *Cxcl9* and *Cxcl10* expression in Bic lentivirus transduced 4T1 (**A**) and AT-3 (**B**) breast cancer cells using qPCR, compared to those transduced by control GFP lentivirus. (**C-D**) Intracellular CXCL9 expression in in vitro cultivated EO771, 4T1 and AT-3-GFP/Bic breast cancer cells by flow cytometry, representative overlapping histograms (**C**) and quantified MFI of CXCL9 are shown (**D**). (**E**) Correlations of miR-155 levels with T cell recruitment related genes in human BRCA tumors. (**A**, **B** and **D**) Statistical significance was assessed using unpaired, two-tailed, Student's *t*-test and all data points are presented as mean ± SEM. $^{**}P < 0.01$, $^{***}P < 0.001$, $^{****}P < 0.0001$. n = 3. *P* and *r* value in (**E**) were calculated based on the Pearson's correlation analysis. n = 995.

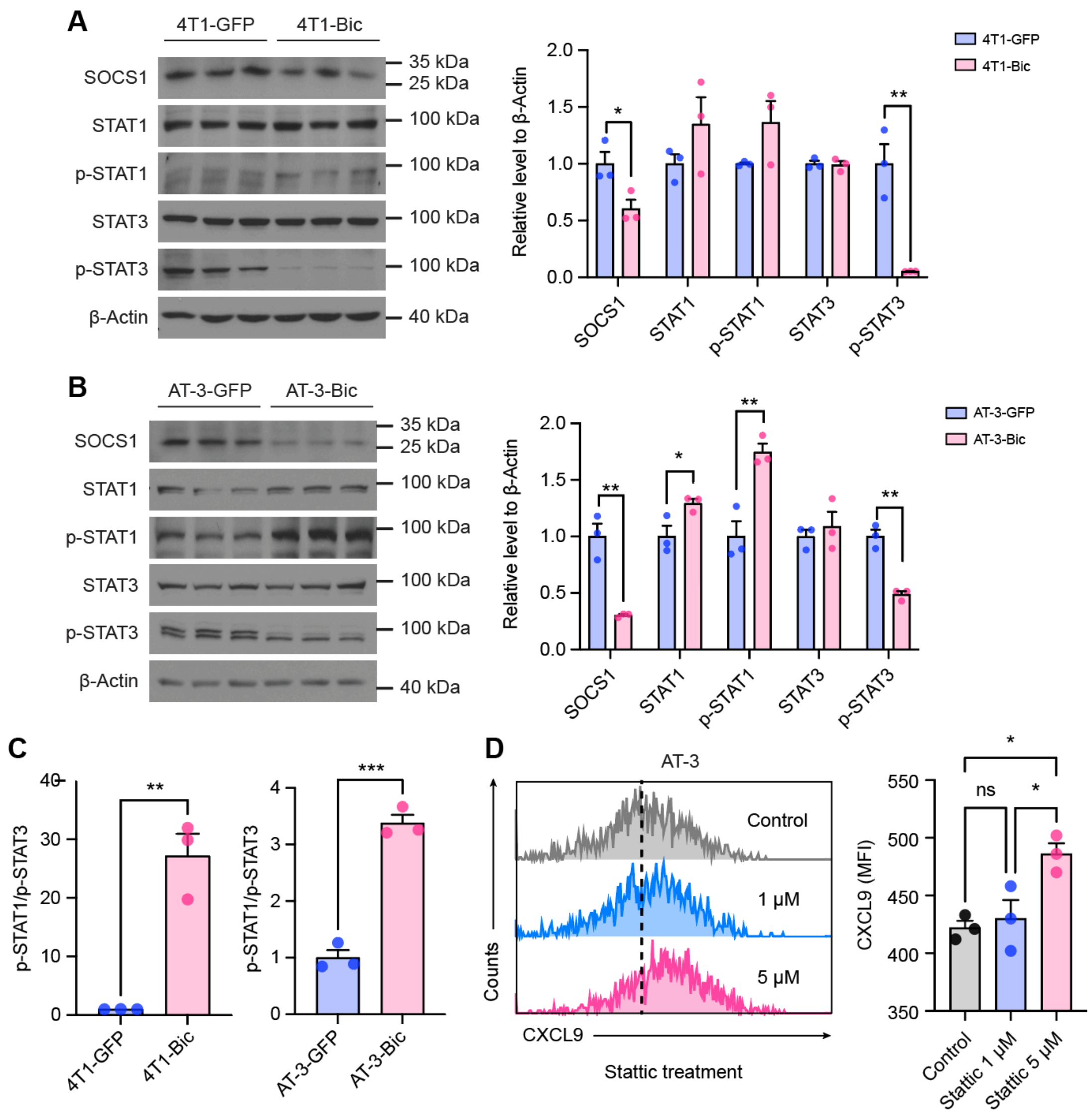

Supplementary Figure 11. miR-155 upregulates CXCL9 expression in 4T1 and AT-3 cells by tilting p-STAT1/p-STAT3 ratio. Representative western blotting showing SOCS1 and STAT1/STAT3 protein levels in 4T1-GFP/Bic (**A**) and AT-3-GFP/Bic (**B**) cells; the bar graphs were generated by quantifying blots relative to β-Actin expression. For samples run on different gels, separate loading controls were provided in Supplemental Unedited Western Blot images. (**C**) The ratio of p-STAT1 to p-STAT3 in 4T1-GFP/Bic cells and AT-3-GFP/Bic cells based on bands intensity. (**D**) Intracellular CXCL9 expression in in AT-3 parental cells 24 h post STAT3 inhibitor (Stattic) treatment. Statistical significance was assessed using two-tailed Student's *t* test for comparing 2 groups (**A**-**C**) and one-way ANOVA followed by Tukey's post-hoc test for multiple groups (**D**). All data points are presented as mean ± SEM. *$P < 0.05$, **$P < 0.01$, ***$P < 0.001$. n = 3 per group.

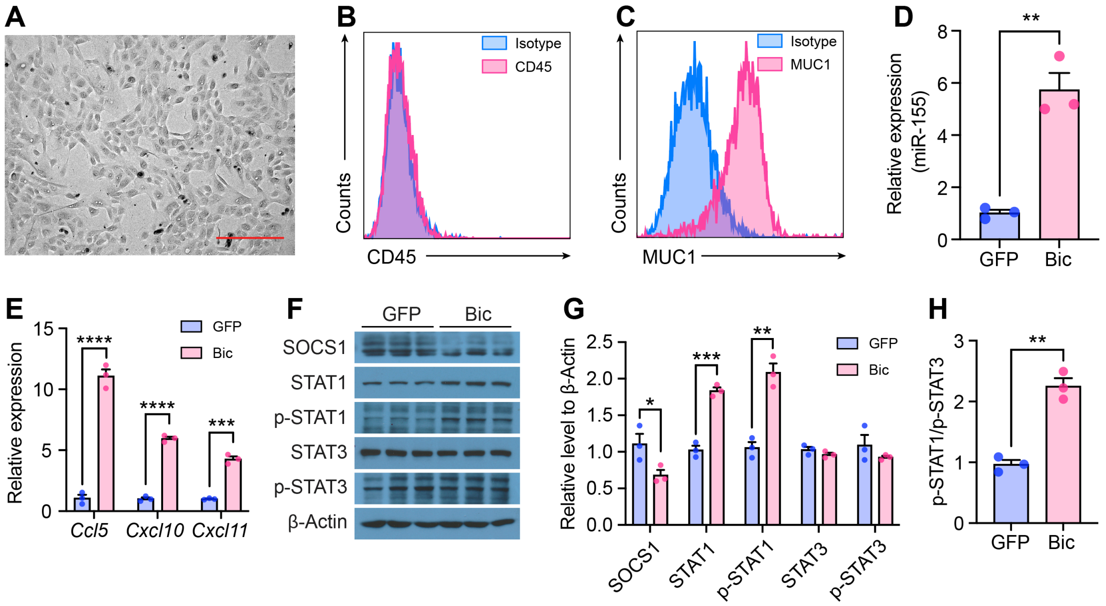

Supplementary Figure 12. Cell intrinsic miR-155 regulates tumor immunity by enhancing the production of chemoattractants in human primary breast cancer cells. (**A**) Human primary breast cancer cell morphology, scale bar: 275 μm. Epithelial cancer identification by determining CD45 (**B**) and MUC1 (**C**) expression on the surface of human primary breast cancer cells. (**D**) Validation of miR-155 expression in GFP and Bic lentiviruses transfected human primary breast cancer cells by qPCR. n = 3 per group. (**E**) *Ccl5* and *Cxcl10/11* expression in GFP and Bic overexpressing human primary breast cancer cells by qPCR. n = 3 per group. (**F**) Representative western blotting bands showing SOCS1 protein, STAT1/STAT3 protein and phosphorylation levels in GFP and Bic lentiviruses transfected human primary breast cancer cells. For samples run on different gels, separate loading controls were provided in Supplemental Unedited Western Blot images. (**G**) Blots of (**F**) was quantified relative to β-Actin expression. n = 3 per group. (**H**) The ratio of p-STAT1 to p-STAT3 in GFP or Bic cells based on band intensity. n = 3 per group. Statistical significance in all figures was assessed using the unpaired, two-tailed, Student's *t*-test. All data points are presented as mean ± SEM. **P* < 0.05, ***P* < 0.01, ****P* < 0.001, *****P* < 0.0001.

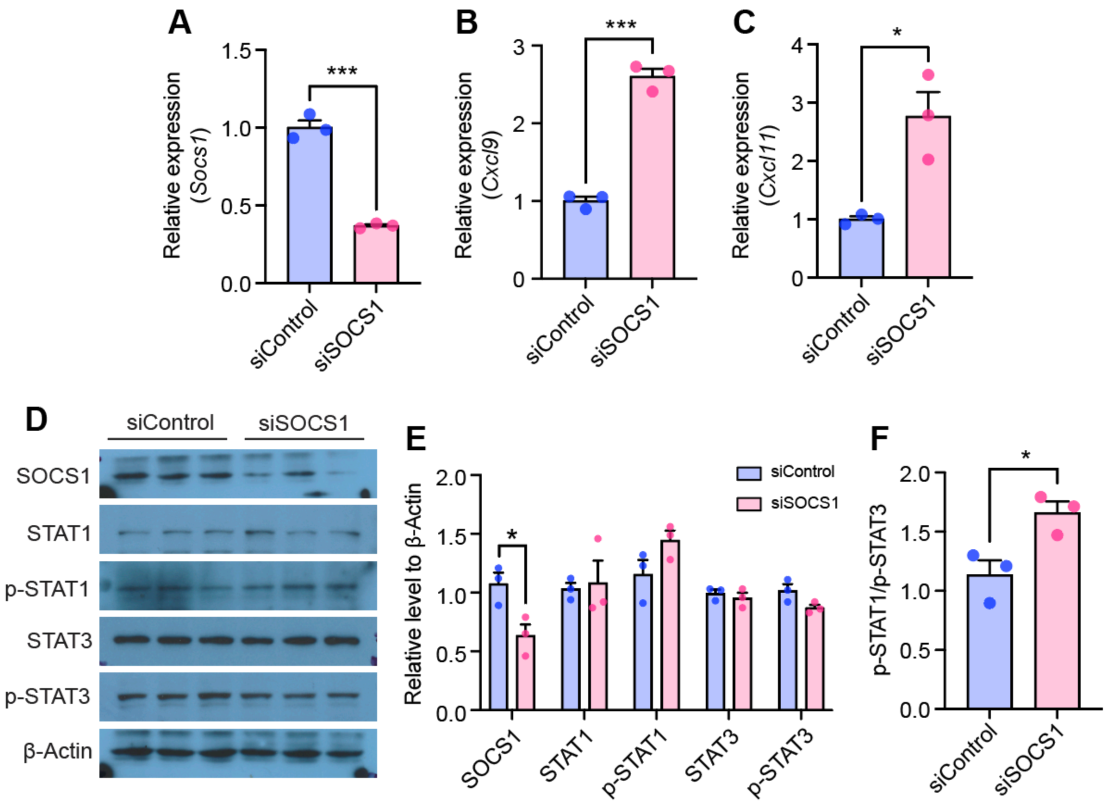

Supplementary Figure 13. SOCS1 silencing in EO771 cells phenocopied miR-155 overexpression. (**A**) *Socs1* expression showing successful SOCS1 knockdown by siRNA targeting SOCS1 in EO771 cells using qPCR. *Cxcl9* (**B**) and *Cxcl11* (**C**) expression in EO771 cells upon SOCS1 siRNA transfection by qPCR. (**D**) Representative western blotting bands showing SOCS1, STAT1/STAT3 protein and phosphorylation levels in EO771 cells transfected with control or siRNAs targeting SOCS1. For samples run on different gels, separate loading controls were provided in Supplemental Unedited Western Blot images. (**E**) Blots were quantified relative to β-Actin expression. (**F**) The ratio of p-STAT1 to p-STAT3 in siControl or siSOCS1 cells based on band intensity. For all experiment, n = 3 per group. Statistical significance was assessed using the unpaired, two-tailed, Student's *t*-test and all data points are presented as mean ± SEM. *$P < 0.05$, **$P < 0.01$, ***$P < 0.001$.

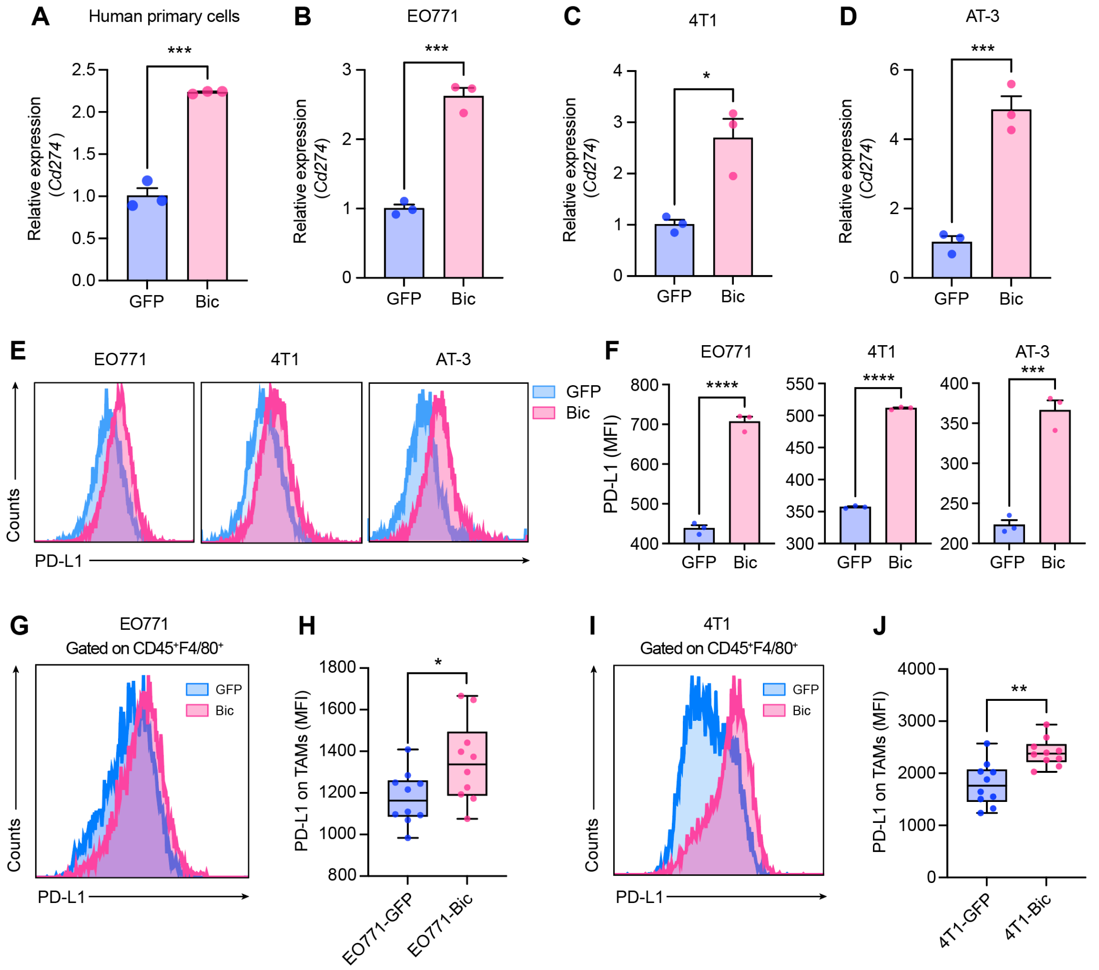

Supplementary Figure 14. **miR-155 upregulates PD-L1 expression in breast cancer cells and TAMs.** PD-L1 (*Cd274*) expression in Bic lentivirus transduced human primary breast cancer (**A**) EO771 (**B**), 4T1 (**C**) and AT-3 cells (**D**) using qPCR, compared to those transduced by control GFP lentivirus. (**E** and **F**) Cell surface PD-L1 expression on cultured GFP/Bic breast cancer cells by flow cytometry, representative overlapping histograms (**E**) and quantified MFI of PD-L1 (**F**) are shown. PD-L1 expression on TAMs retrieved from EO771 (**G** and **H**) and 4T1 (**I** and **J**) tumor tissues; the representative histogram plots and quantified MFI of PD-L1 are shown, respectively. For **A**-**F**, n = 3 per group; **G**-**J**, n = 10 per group. Statistical significance was assessed using the unpaired, two-tailed, Student's *t*-test and all data points are presented as mean ± SEM. *$P < 0.05$, **$P < 0.01$, ***$P < 0.001$, ****$P < 0.0001$.

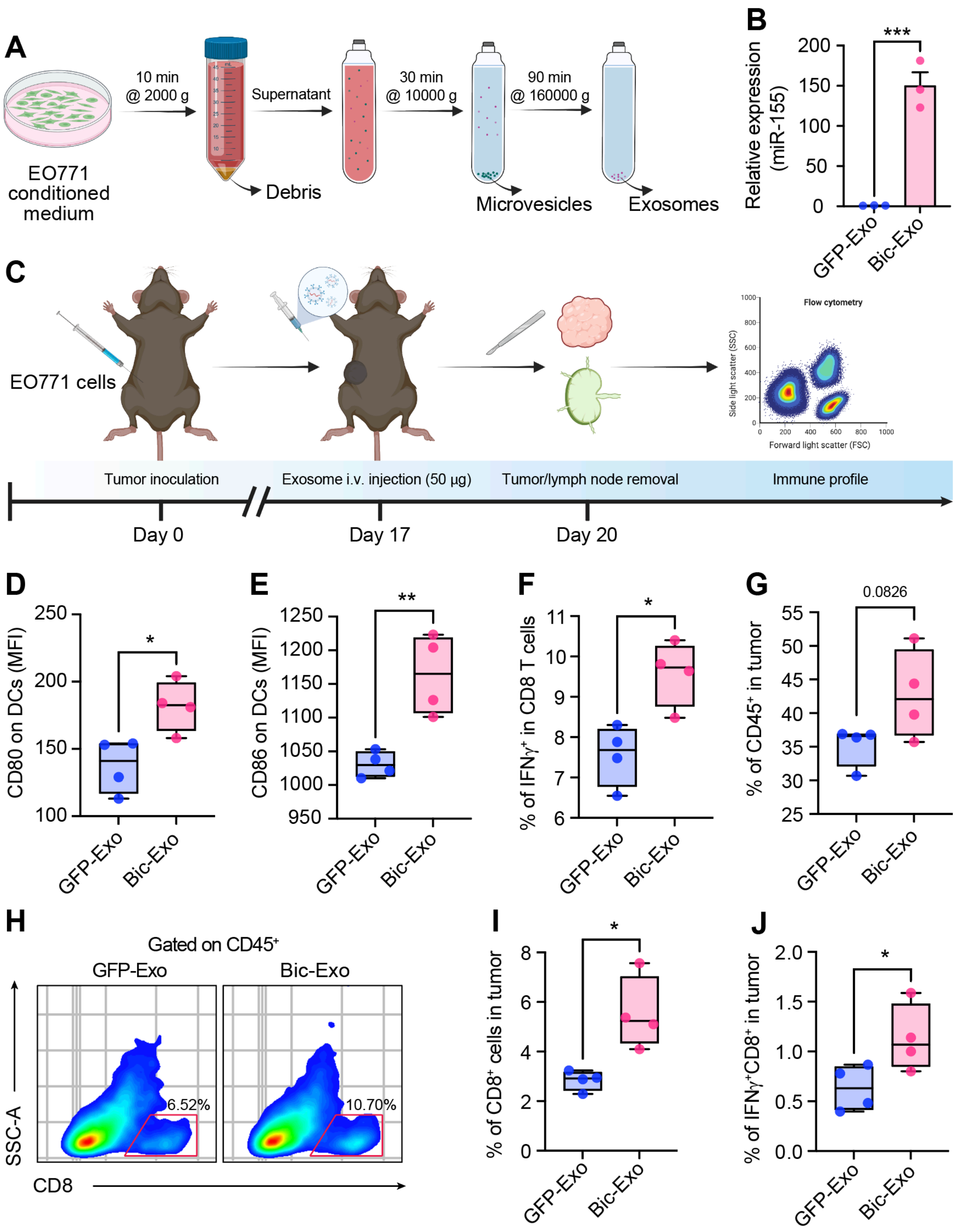

Supplementary Figure 15. Tumor-derived exosomes containing miR-155 enhance anti-tumor immune response. (**A**) Schematic image illustrating the procedure of exosome collection from tumor conditioned medium. (**B**) miR-155 expression in exosomes collected from GFP and Bic tumor conditioned media, by qPCR. n = 3 per group. (**C**) Schematic image illustrating the workflow of the in vivo study. CD80 (**D**) and CD86 (**E**) levels on CD11c$^+$ dendritic cells of lymph nodes. (**F**) Percentages of IFNγ$^+$ producing CD8$^+$ T cells in lymph nodes. (**G**) Frequencies of tumor infiltrating CD45$^+$ leukocytes. n = 4 per group. (**H**) The representative pseudo color images from 4 samples of each group showing the frequencies of CD8$^+$ T cells gating from CD45$^+$ cells. Quantified percentage of CD8$^+$ (**I**) and IFNγ$^+$CD8$^+$ (**J**) T cells in tumors. For figure **D-J**, n = 4 per group. Statistical significance was assessed using

the unpaired, two-tailed, Student's *t*-test and all data points are presented as mean ± SEM. *$P < 0.05$, **$P < 0.01$, ***$P < 0.001$.

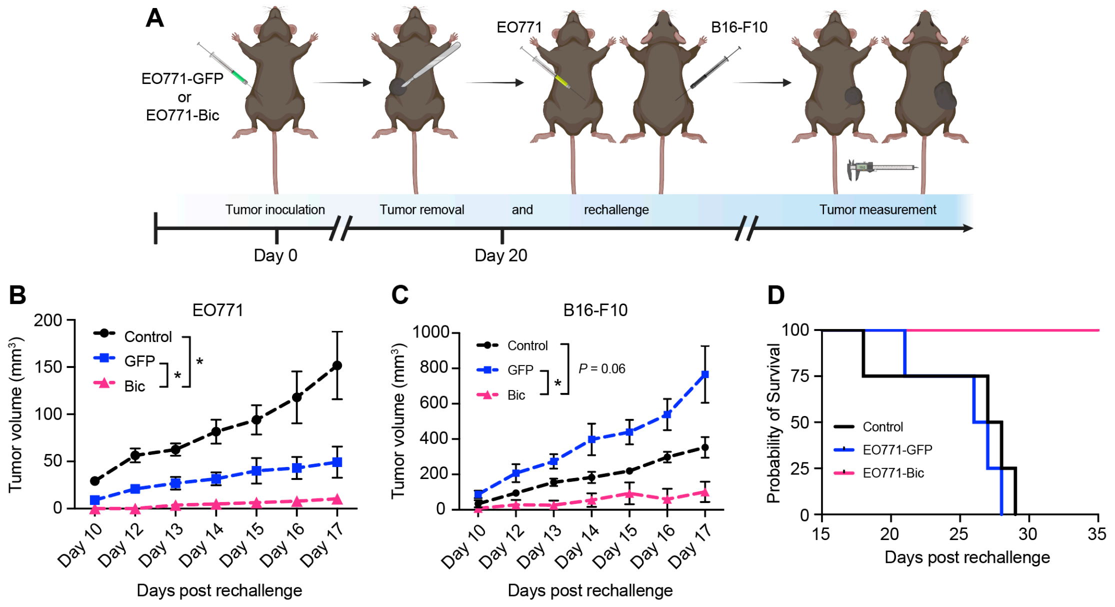

Supplementary Figure 16. Breast tumors with miR-155-overexpressing breast cancer cells trigger stronger immune memory. (**A**) Schematic image illustrating the mouse experimental design of immune memory study. After primary tumor removal on Day 20, the dynamic tumor growth curves of rechallenged EO771 (**B**) and B16-F10 (**C**) tumors are shown. n = 4 per group. (**D**) Survival probability of mice rechallenged with EO771 and B16-F10 tumor cells. Statistical analysis of (**B**) and (**C**) was performed using two-way analysis of variance followed by Tukey test. All data points are presented as mean ± SEM. *$P < 0.05$.

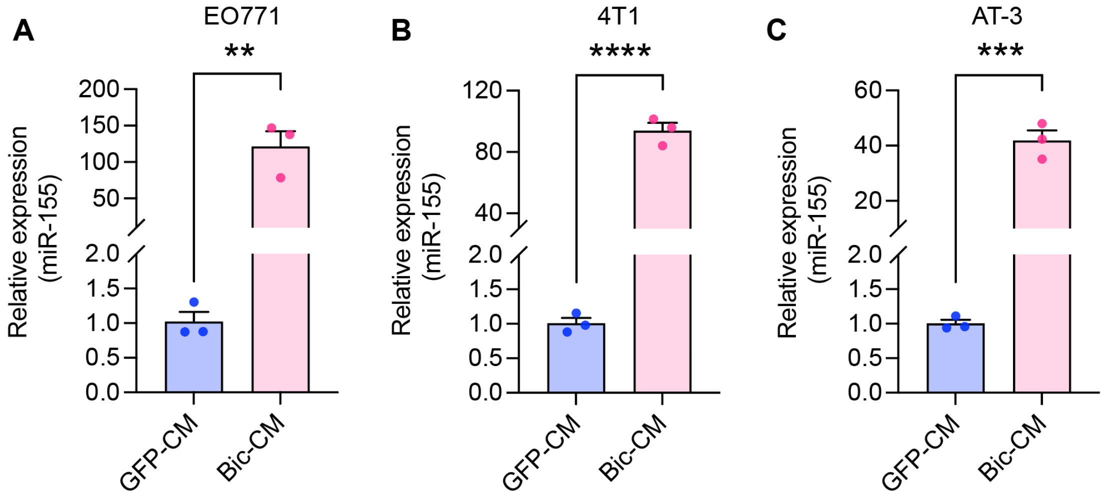

Supplementary Figure 17. **miR-155 expression in tumor conditioned media.** Tumor conditioned media were collected form GFP and Bic lentivirus transduced tumor cells. miR-155 expression in tumor culture media was determined using qPCR. Statistical significance was assessed using the unpaired, two-tailed, Student's *t*-test and all data points are presented as mean ± SEM. ***P* < 0.01; ****P* < 0.001; *****P* < 0.0001. n = 3 per group.

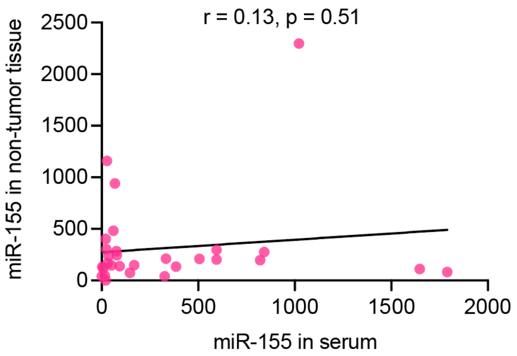

Supplementary Figure 18. **The correlation between serum miR-155 level with non-tumor tissue miR-155 expression.** *P* and r value in was calculated based on the Pearson's correlation analysis. n = 29.

Supplementary tables

Supplementary Table 1. Clinical characteristics of 29 patients with BRCA

Characteristic	Patient #	Patient %
Age, years (≤ 50/> 50)	7/22	24.1/75.9
Sex (male/female)	0/29	0/100
Tumor size, cm (≤ 3/> 3)	19/10	65.5/34.5
Tumor differentiation (Ⅰ-Ⅱ/Ⅲ-Ⅳ)	10/19	34.5/65.5
TNM stage (Ⅰ-II/III)	19/10	65.5/34.5

Supplementary Table 2. Multivariate survival analysis

Variables	HR	95% CI	*P* value
Age	1.043	[1.027, 1.058]	0
Gender Male (ref.=Female)	0.590	[0.081, 4.322]	0.603
Race Asian (ref.=White)	1.095	[0.264, 4.549]	0.9
Race Black (ref.=White)	1.208	[0.710, 2.056]	0.486
Pathologic stage I-II	1.941	[1.051, 3.587]	0.034*
Pathologic stage III-IV	4.635	[2.485, 8.648]	0
Tumor purity	0.116	[0.021, 0.647]	0.014*
Hsa-miR-155 expression	0.724	[0.544, 0.965]	0.028*

Abbreviation: HR, hazard ratio; CI, confidence interval.
* Statistically significant with *P* value < 0.05.

Supplementary Table 3. List of most significant upregulated genes in miR-155^{high} tumors

Order	Gene ID	log2 Fold Change	P-adj	Order	Gene ID	log2 Fold Change	P-adj
1	*MS4A1*	3.663822007	9.88E-14	33	*SLA2*	2.296290082	3.67E-12
2	*PLA2G2D*	3.535850865	5.68E-32	34	*CD52*	2.279673223	0.02089843
3	*CR2*	3.30541749	8.81E-06	35	*ZNF683*	2.2661227	2.48E-13
4	*CD38*	3.261727586	1.84E-14	36	*CCL18*	2.252438751	2.68E-22
5	*CD79A*	2.929180651	7.88E-34	37	*IDO1*	2.237723128	8.99E-08
6	*SIRPG*	2.882926416	8.48E-19	38	*CXCR3*	2.221301451	1.69E-35
7	*CXCL9*	2.837206611	1.01E-67	39	*SIT1*	2.219864875	4.58E-27
8	*GZMB*	2.823032585	9.37E-48	40	*CD3D*	2.211602124	9.81E-57
9	*POU2AF1*	2.75322476	4.98E-20	41	*S1PR4*	2.195362845	6.68E-16
10	*AIM2*	2.748844602	1.66E-15	42	*OR2I1P*	2.193813564	4.96E-21
11	*TIGIT*	2.726647792	2.38E-18	43	*CCL13*	2.193565057	7.16E-20
12	*PDCD1*	2.71409189	6.40E-13	44	*CXCL11*	2.183237953	4.41E-32
13	*SH2D1A*	2.691017151	1.37E-22	45	*MARCO*	2.182148057	2.07E-12
14	*CTLA4*	2.68602313	4.60E-15	46	*NKG7*	2.181454085	9.75E-50
15	*VPREB3*	2.681036138	1.24E-14	47	*CD27*	2.16711725	4.59E-50
16	*SPIB*	2.626712571	2.52E-06	48	*CD79B*	2.166786646	1.62E-21
17	*IGLL5*	2.614995126	1.03E-21	49	*CD3E*	2.163700178	6.45E-58
18	*GPR171*	2.569770518	2.46E-11	50	*UBD*	2.147051025	8.08E-18
19	*LAX1*	2.554150892	8.70E-12	51	*CD96*	2.144142677	7.36E-17
20	*GBP5*	2.472148211	6.49E-36	52	*CXCR6*	2.144030023	1.78E-11
21	*PRKCB*	2.465407155	2.31E-09	53	*GZMM*	2.132155783	3.03E-22
22	*IRF4*	2.457690767	4.60E-15	54	*CD5*	2.130600858	2.58E-32
23	*CHIT1*	2.45477172	0.01652543	55	*CD247*	2.124602024	1.43E-16
24	*SLAMF6*	2.429652582	2.89E-34	56	*CD2*	2.122873859	1.20E-71
25	*TNFRSF17*	2.426095121	2.59E-22	57	*ADAMDEC1*	2.105373447	4.81E-31
26	*ZAP70*	2.419654928	4.61E-14	58	*CCL5*	2.103814659	1.11E-52
27	*LTB*	2.407438214	1.04E-14	59	*SLAMF7*	2.090886194	9.37E-48
28	*KLHDC7B*	2.371994217	1.13E-09	60	*LCK*	2.069531601	1.80E-36
29	*XCL2*	2.370333504	2.26E-09	61	*IL2RA*	2.065184511	5.21E-12
30	*GZMK*	2.343769967	3.03E-49	62	*HLA-DOB*	2.025233425	2.31E-25
31	*CCL19*	2.34126995	1.01E-26	63	*CD7*	2.020362659	9.56E-23
32	*CD3G*	2.300752619	1.07E-13	64	*CD8B*	2.014532382	5.03E-19

Supplementary Table 4. Antibodies used in flow cytometry

Antibody-Fluorescein	Catalog number	Clone number	Manufacturer
CD45-PE	103106	30-F11	Biolegend
CD45-Percp/Cy5.5	103132	30-F11	Biolegend
CD3-APC-Cy7	100330	145-2C11	Biolegend
CD4-APC	100412	GK1.5	Biolegend
CD8-Percp-Cy5.5	100733	53-6.7	Biolegend
PD-L1-APC	124311	10F.9G2	Biolegend
IFNγ-APC-Cy7	505849	XMG1.2	Biolegend
FOXP3-APC	51-5773-82	FJK-16s	eBioscience
CD11c-APC	117310	N418	Biolegend
F4/80-FITC	123108	BM8	Biolegend
CXCL9-PE	515603	MIG-2F5.5	Biolegend
CCR5-APC	107012	HM-CCR5	Biolegend
CXCR3-PE-Cy7	126515	CXCR3-173	Biolegend
NK1.1-PE	108707	PK136	Biolegend
Brdu-PE-Cy7	364118	3D4	Biolegend
MUC-1-PE-Cy7	355605	16A	Biolegend

Supplementary Table 5. Primer sequences used in qPCR gene expression analysis

Gene	Forward primer (5'-3')	Reverse primer (5'-3')
mCd2	GATGAGAAACGACAGTGGCACC	CCAGTGGATCATGGGCTTTGAG
mCd8a	ACTACCAAGCCAGTGCTGCGAA	ATCACAGGCGAAGTCCAATCCG
mIl2	GCGGCATGTTCTGGATTTGACTC	CCACCACAGTTGCTGACTCATC
mIl2rg	GGAGCAACAGAGATCGAAGCTG	CCACAGATTGGGTTATAGCGGC
mGzmb	CAGGAGAAGACCCAGCAAGTCA	CTCACAGCTCTAGTCCTCTTGG
mSlamf6	CAGAGCCAAGAATGCTGTCAGC	ACCATACTGCATTCCAGGGTGG
mIfng	CAGCAACAGCAAGGCGAAAAAGG	TTTCCGCTTCCTGAGGCTGGAT
mTnfa	GGTGCCTATGTCTCAGCCTCTT	GCCATAGAACTGATGAGAGGGAG
mCcl5	TGCCCACGTCAAGGAGTATTTC	AACCCACTTCTTCTCTGGGTTG
mCxcl9	CCTAGTGATAAGGAATGCACGATG	CTAGGCAGGTTTGATCTCCGTTC
mCxcl10	ATCATCCCTGCGAGCCTATCCT	GACCTTTTTTGGCTAAACGCTTTC
mCxcl11	CCGAGTAACGGCTGCGACAAAG	CCTGCATTATGAGGCGAGCTTG
mPdcd1	CGGTTTCAAGGCATGGTCATTGG	TCAGAGTGTCGTCCTTGCTTCC
mCD274	TGCGGACTACAAGCGAATCACG	CTCAGCTTCTGGATAACCCTCG
mCtla4	GTACCTCTGCAAGGTGGAACTC	CCAAAGGAGGAAGTCAGAATCCG
mTigit	CCACAGCAGGCACGATAGATA	CATGCCACCCCAGGTCAAC
mLag3	CTCCATCACGTACAACCTCAAGG	GGAGTCCACTTGGCAATGAGCA
mIl10	CGGGAAGACAATAACTGCACCC	CGGTTAGCAGTATGTTGTCCAGC
mFoxp3	CTCGCATGTTCGCCTACTTC	GGGATTGGAGCACTTGTTGG
mIdo1	AGGATGCGTGACTTTGTGGA	TCCCAGACCCCCTCATACAG
18s	CGCGGTTCTATTTTGTTGGT	AGTCGGCATCGTTTATGGTC
hCD8A	ACTTGTGGGGTCCTTCTCCTGT	TGTCTCCCGATTTGACCACAGG
hIL2	AGAACTCAAACCTCTGGAGGAAG	GCTGTCTCATCAGCATATTCACAC
hIFNG	GAGTGTGGAGACCATCAAGGAAG	TGCTTTGCGTTGGACATTCAAGTC
hCD274	TGCCGACTACAAGCGAATTACTG	CTGCTTGTCCAGATGACTTCGG
hCXCL10	GGTGAGAAGAGATGTCTGAATCC	GTCCATCCTTGGAAGCACTGCA

hCXCL11	AAGGACAACGATGCCTAAATCCC	CAGATGCCCTTTTCCAGGACTTC
hCCL5	CCTGCTGCTTTGCCTACATTGC	ACACACTTGGCGGTTCTTTCGG
hGAPDH	GTCTCCTCTGACTTCAACAGCG	ACCACCCTGTTGCTGTAGCCAA

Supplementary Table 6. Antibodies used in western blotting

Antibody	Catalog number	Clone number	Manufacturer	Dilution
SOCS1	GTX100657	---	GeneTex	1: 1000
p-STAT1	9167	58D6	Cell Signaling Technology	1: 3000
STAT1	sc-346	E-23	Santa Cruz	1: 3000
p-STAT3	9145	D3A7	Cell Signaling Technology	1: 3000
STAT3	sc-482	C-20	Santa Cruz	1: 3000
β-Actin	A2066	---	Sigma	1: 6000-10000

Unedited Western Blot Images

Figure 5I

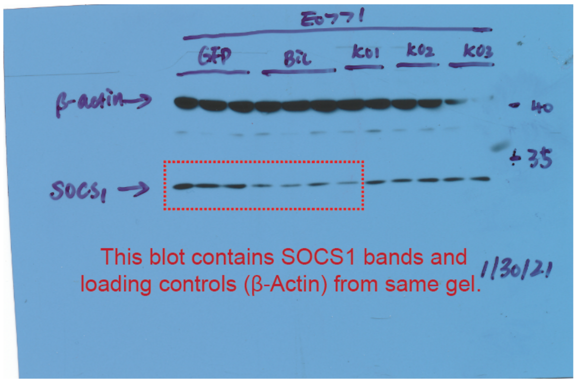

Figure 5I, SOCS1, GeneTex (GTX100657), 1:1000

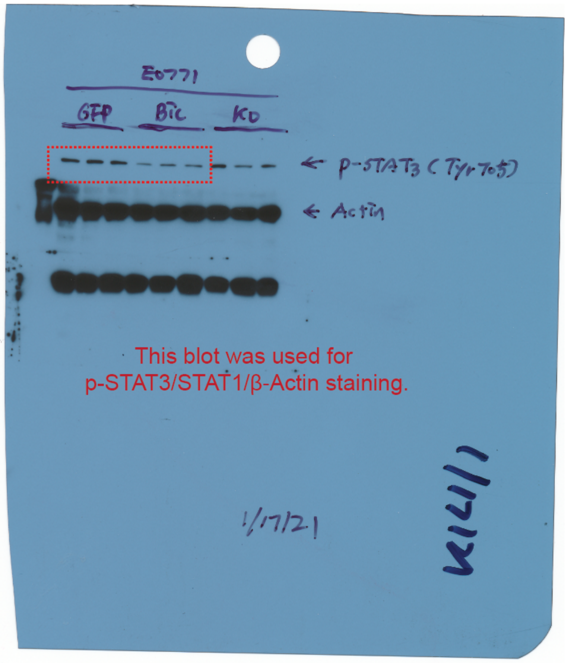

Figure 5I, p-STAT3
Cell Signaling Technology (9145), 1:3000

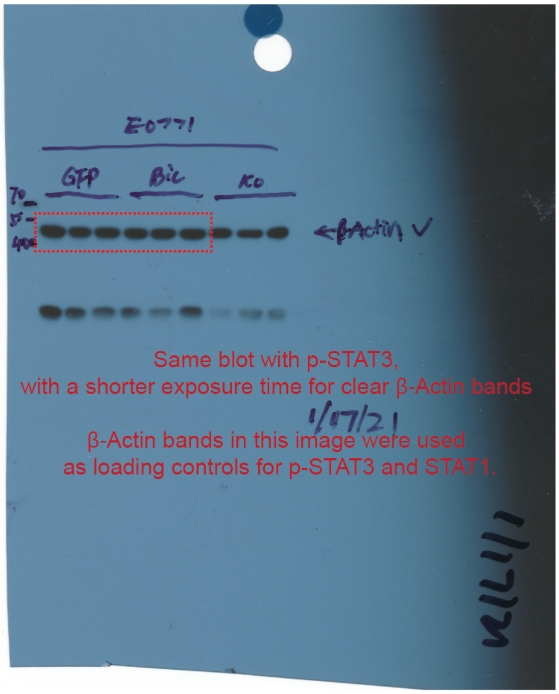

Figure 5I, β-Actin, Sigma (A2066), 1:6000

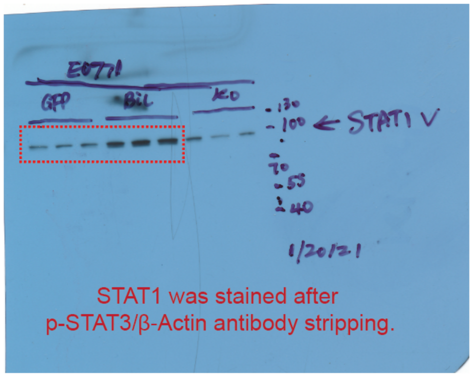

Figure 5I, STAT1, Santa Cruz (sc-346), 1:3000

Note: miR155^{KO} cells were not well established at this time, so we didn't see reversed results as of Bic smaples .

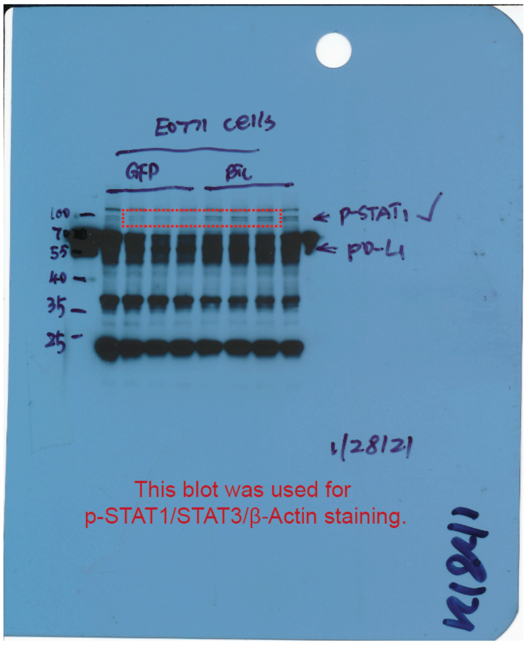

Figure 5I, p-STAT1
Cell Signaling Technology (9167), 1:3000

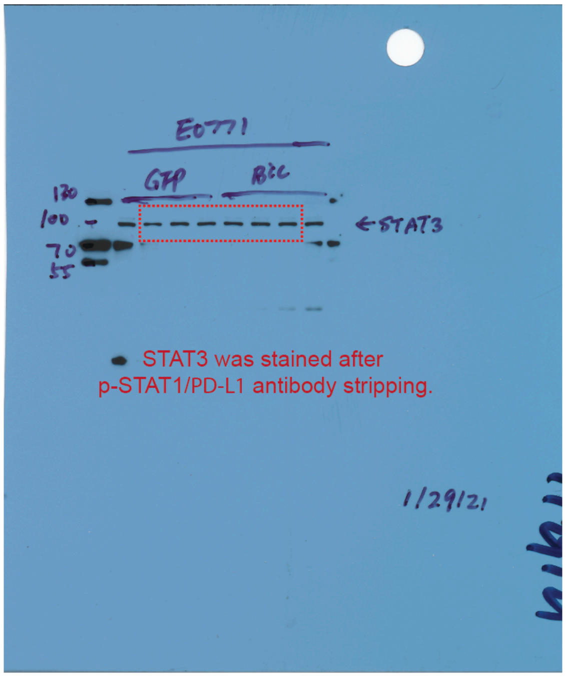

Figure 5I, STAT3, Santa Cruz (sc-482), 1:3000

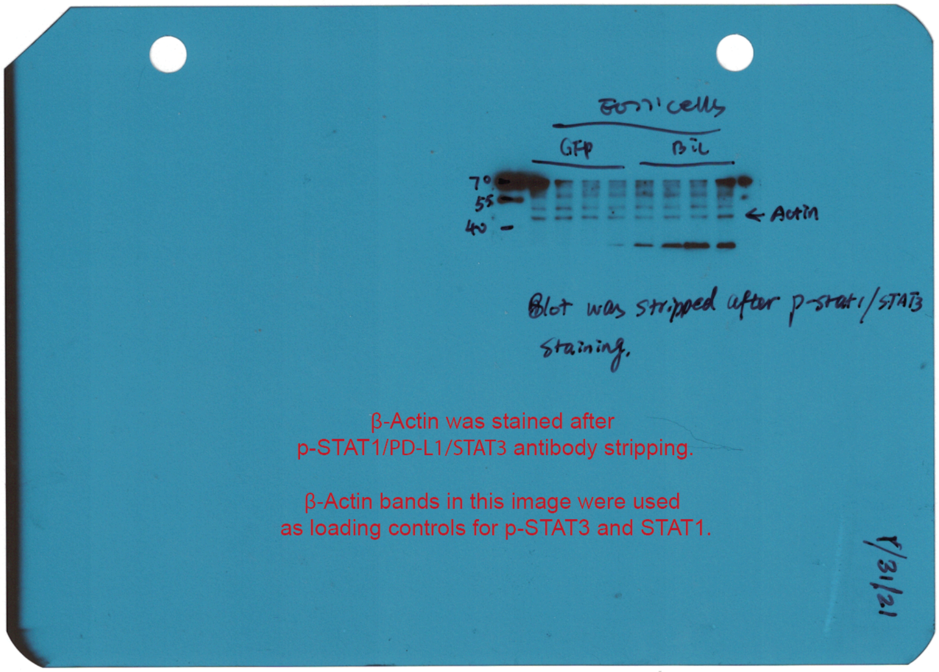

Figure 6M

For Western blot reults in Figure 6M, we loaded samples in seperated gels at the same time (3/12/2022), except for p-STAT3 (same sample aliquotes on 5/3/2022). Each blot had β-Actin as loading controls, as indated below.

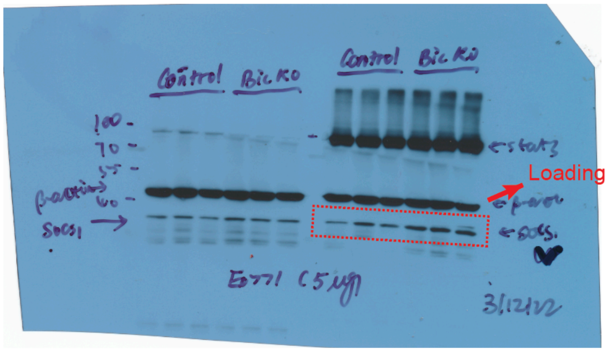

Figure 6M, SOCS1, GeneTex (GTX100657), 1:1000

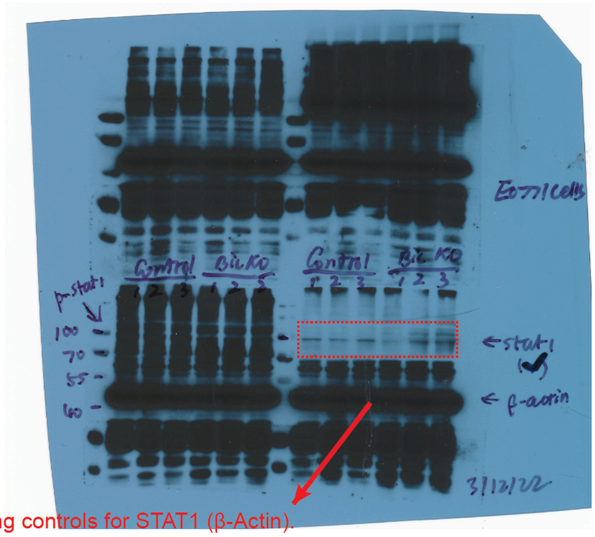

Figure 6M, STAT1, Santa Cruz (sc-346), 1:3000

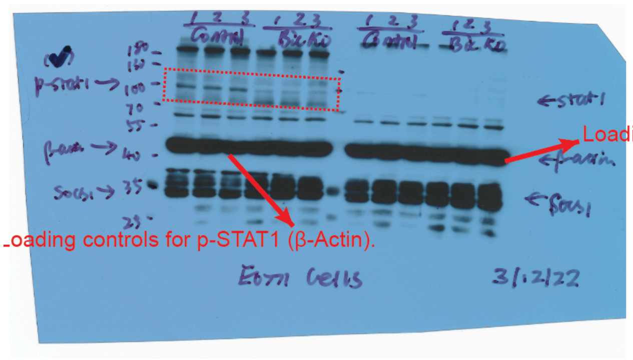

Figure 6M, p-STAT1
Cell Signaling Technology (9167), 1:3000

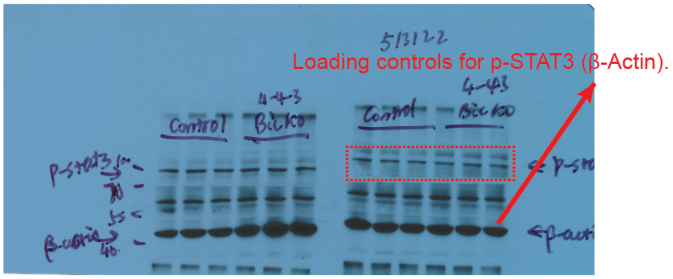

Figure 6M, p-STAT3
Cell Signaling Technology (9145), 1:3000

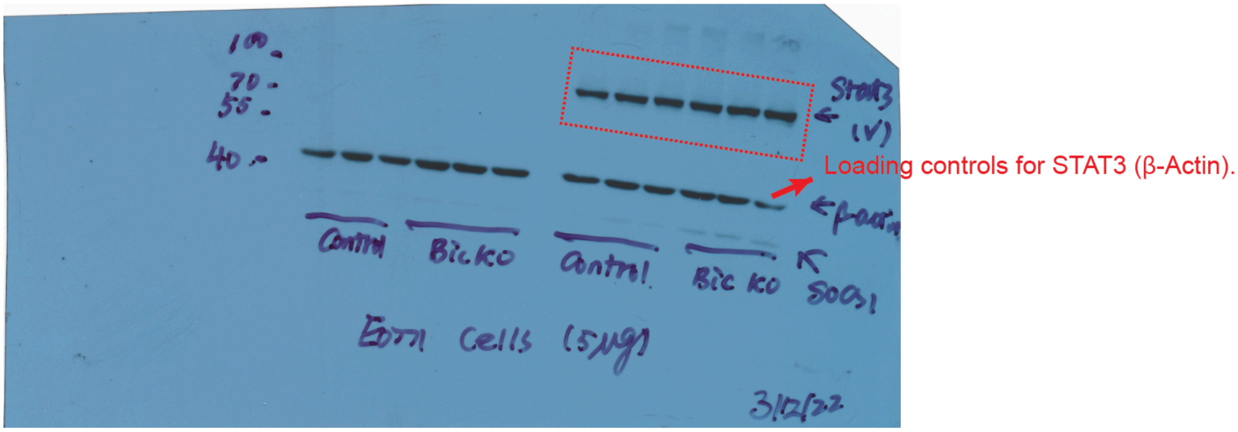

Figure 6M, STAT3, Santa Cruz (sc-482), 1:3000

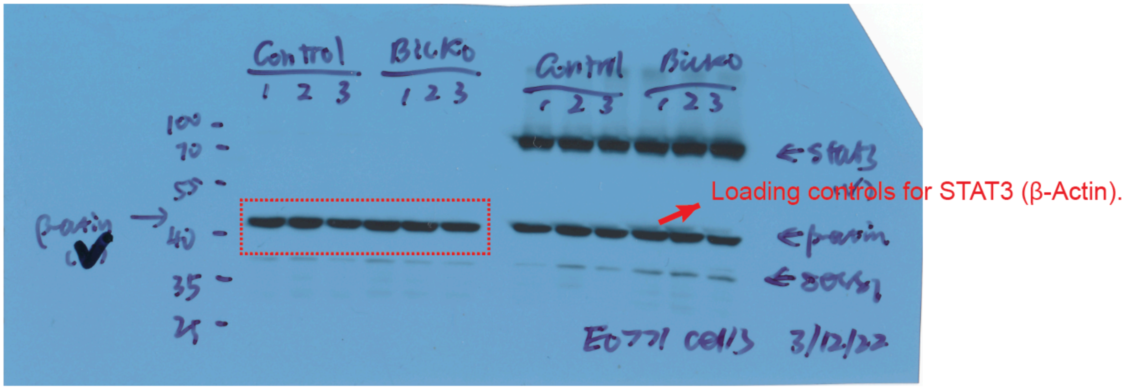

Figure 6M, β-Actin, Sigma (A2066), 1:6000

Supplementary Figure 11A

For Western blot reults in Supplementary Figure 11 A, we loaded samples in seperated gels at the same time (10/09/2021).
Each blot had corresponding β-Actin as loading controls, as indated below.

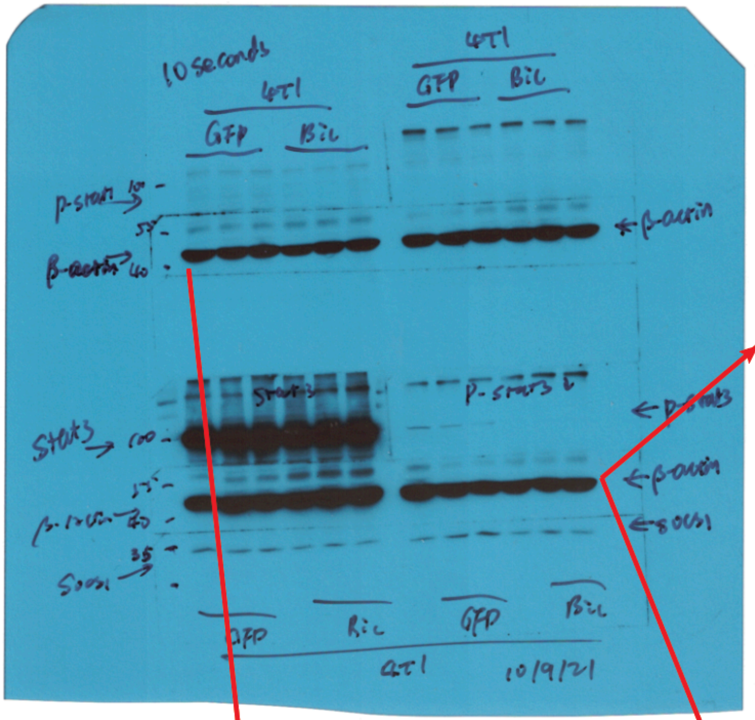

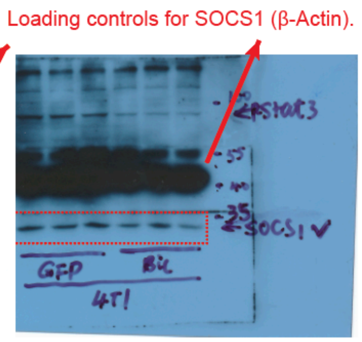

Supplementary Figure 11A, SOCS1
GeneTex (GTX100657), 1:1000

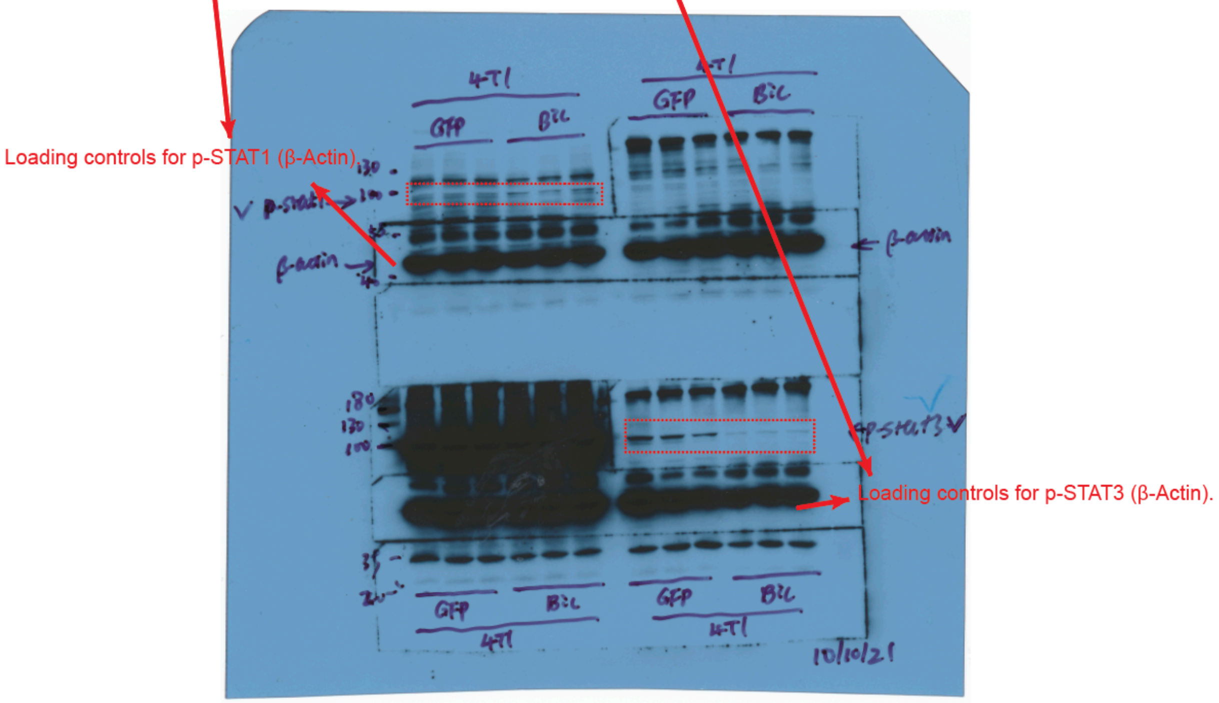

Supplementary Figure 11A, p-STAT1
Cell Signaling Technology (9167), 1:3000

Supplementary Figure 11 A, p-STAT3
Cell Signaling Technology (9145), 1:3000

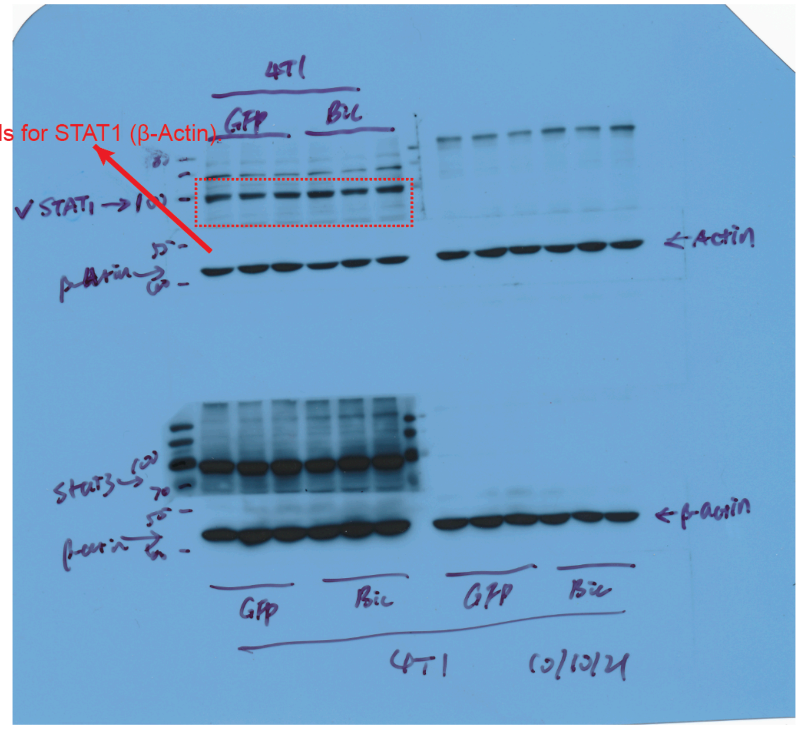

Supplementary Figure 11A, STAT1
Santa Cruz (sc-346), 1:3000

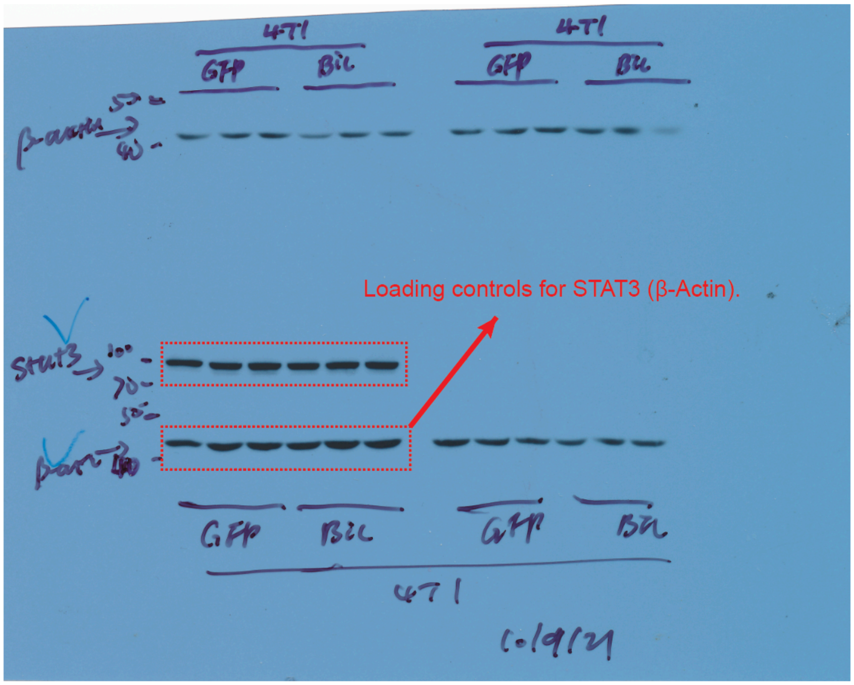

Supplementary Figure 11A, STAT3
Santa Cruz (sc-482), 1:3000

Supplementary Figure 11A, β-Actin
Sigma (A2066), 1:6000

Supplementary Figure 11B

For Western blot reults in Supplementary Figure 11 B, we loaded samples in seperated gels at the same time 08/18/2021).
Each blot had corresponding β-Actin as loading controls, as indated below.

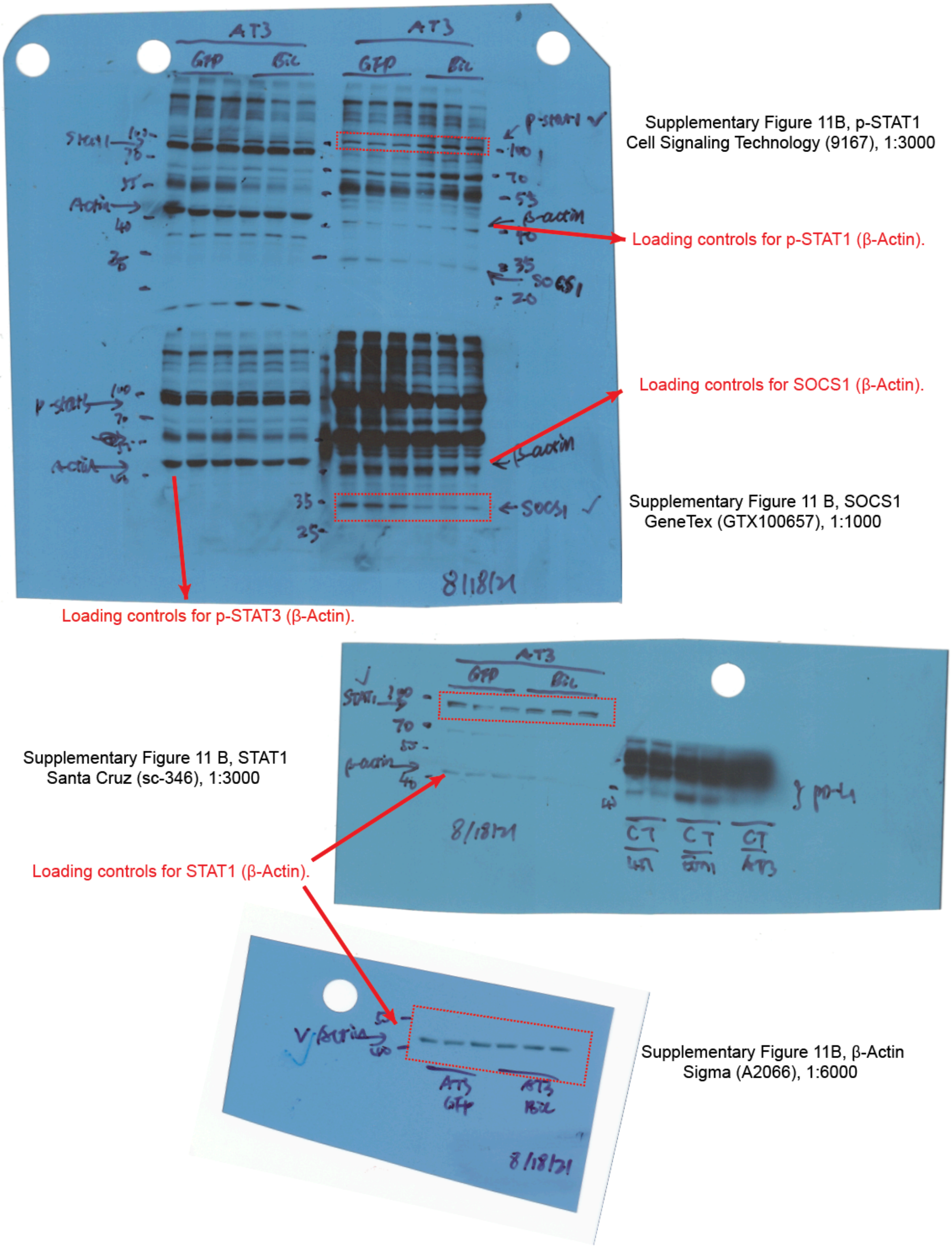

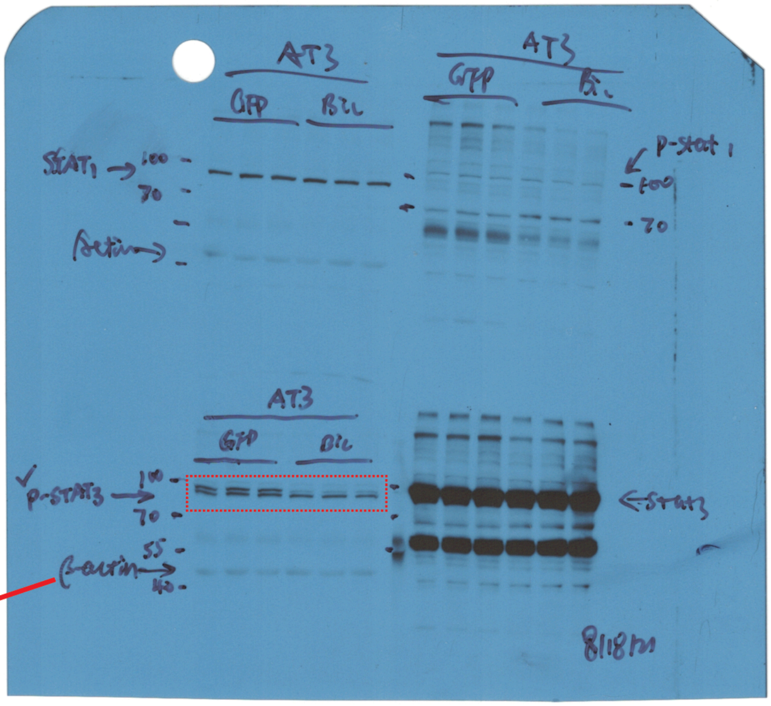

Supplementary Figure 11B, p-STAT3
Cell Signaling Technology (9145), 1:3000

Loading controls for p-STAT3 (β-Actin).

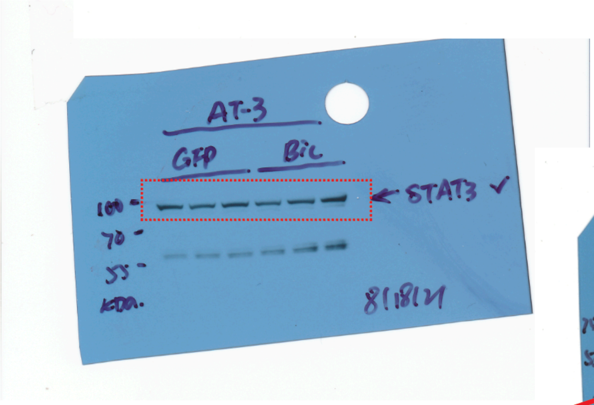

Supplementary Figure 11B, STAT3
Santa Cruz (sc-482), 1:3000

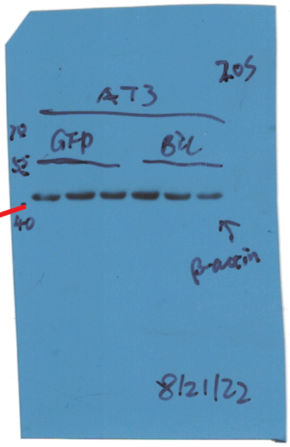

Loading controls for STAT3 (β-Actin).
Blot was stripped and restained with β-Actin antibody.

Supplementary Figure 12F

For Western blot reults in Supplementary Figure 12F, we loaded the aliquotes of samples in seperated gels at different time (05/03/2022 and 05/12/2022). Each blot had corresponding β-Actin as loading controls, as indated below.

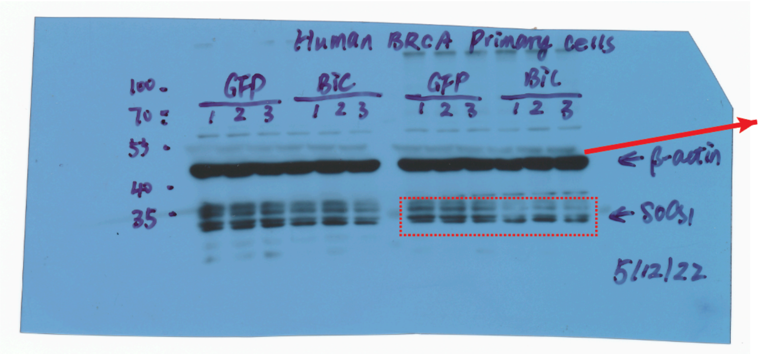

Loading controls for SOCS1 (β-Actin).

Supplementary Figure 12F,
SOCS1, GeneTex (GTX100657), 1:1000

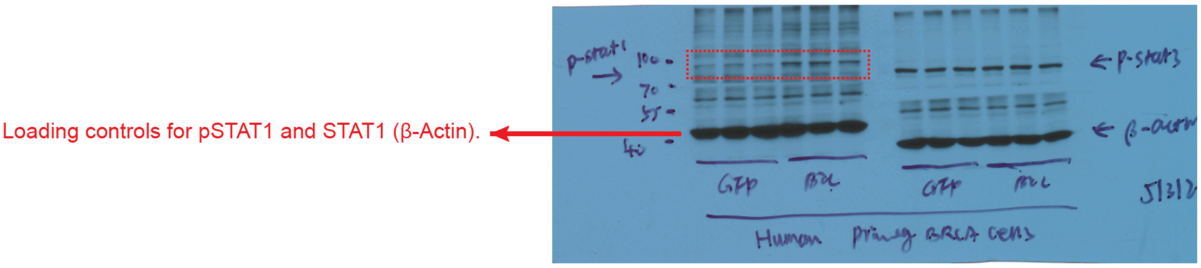

Loading controls for pSTAT1 and STAT1 (β-Actin).

Supplementary Figure 12F, p-STAT1
Cell Signaling Technology (9167), 1:3000

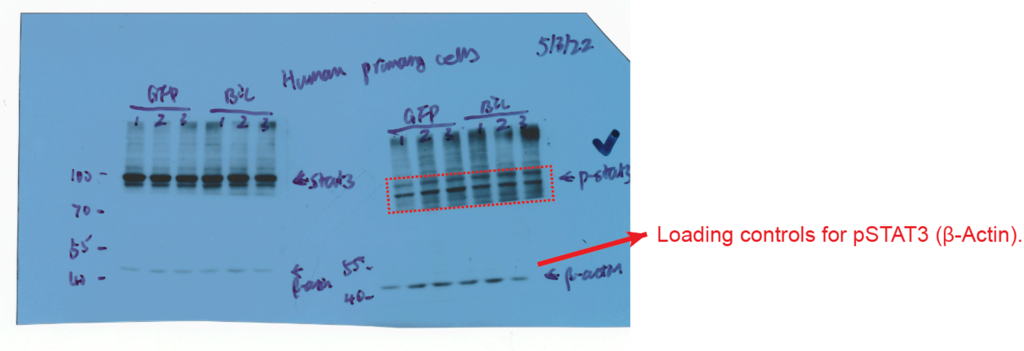

Loading controls for pSTAT3 (β-Actin).

Supplementary Figure 12F, p-STAT3
Cell Signaling Technology (9145), 1:3000

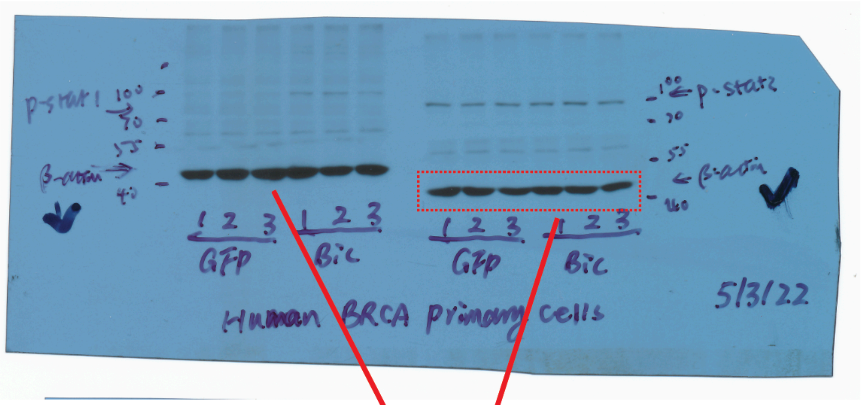

Supplementary Figure 12F,
β-Actin, Sigma (A2066), 1:6000

Above blots were stripped and
restained with STAT1 and STAT3 antibodies, respectively.

Loading controls for STAT3 (β-Actin).

Loading controls for STAT1 (β-Actin).

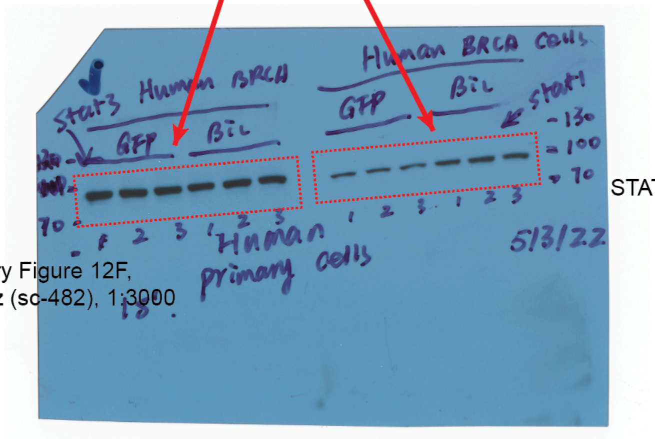

Supplementary Figure 12F,
STAT1, Santa Cruz (sc-346), 1:3000

Supplementary Figure 12F,
STAT3, Santa Cruz (sc-482), 1:3000

Supplementary Figure 13D

For Western blot reults in Supplementary Figure 13D, we loaded samples in seperated gels.
Each blot had corresponding β-Actin as loading controls, as indated below.

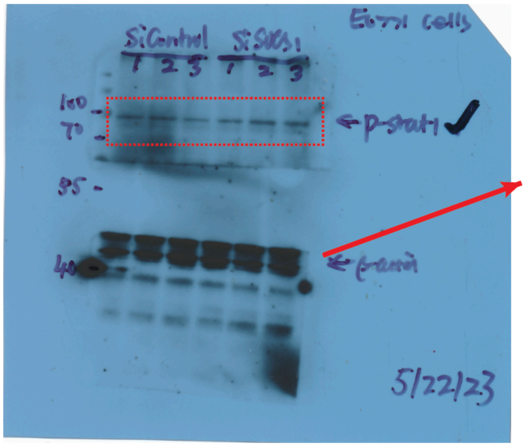

Loading controls for p-STAT1 (β-Actin).

Supplementary Figure 13D, p-STAT1
Cell Signaling Technology (9167), 1:3000

Loading controls for STAT1 (β-Actin).

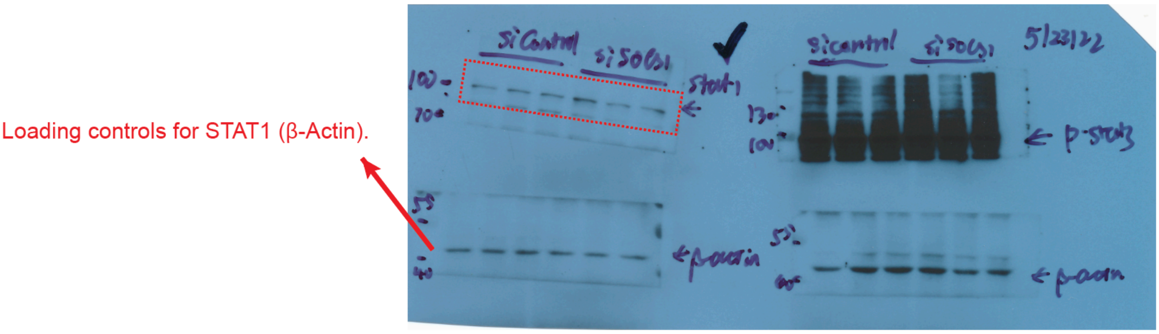

Supplementary Figure 13D,
STAT1, Santa Cruz (sc-346), 1:3000

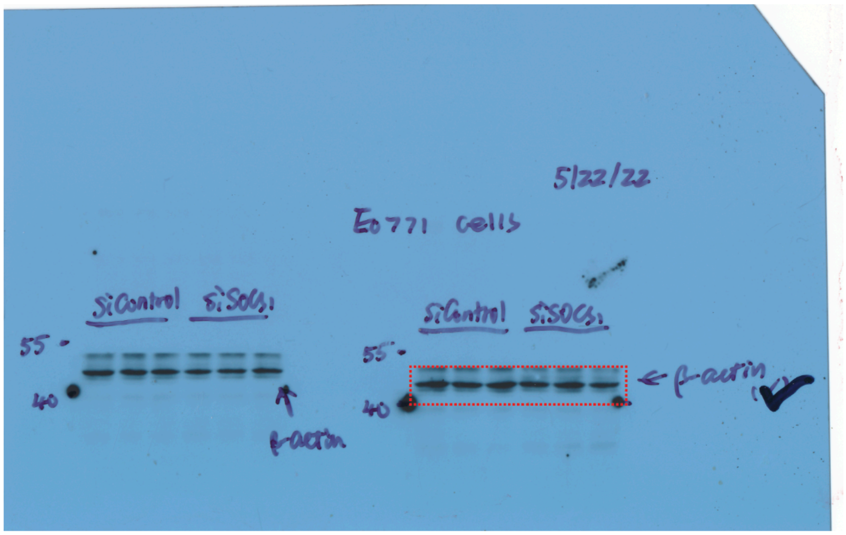

Supplementary Figure 13D, β-Actin, Sigma (A2066), 1:6000

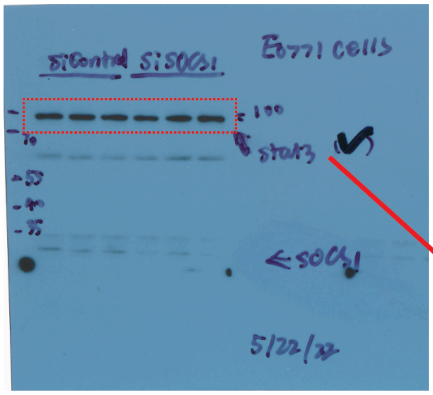

Same blot

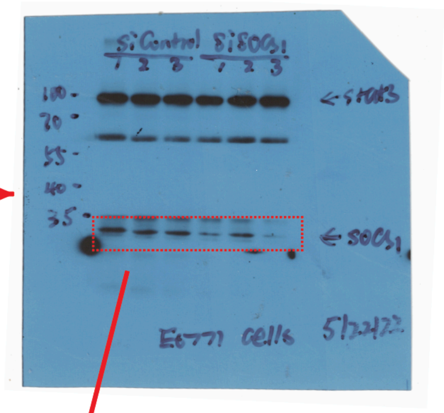

Supplementary Figure 13D, STAT3, Santa Cruz (sc-482), 1:3000

Supplementary Figure 13D,
SOCS1, GeneTex (GTX100657), 1:1000

Blot was stripped and restained with β-Actin.

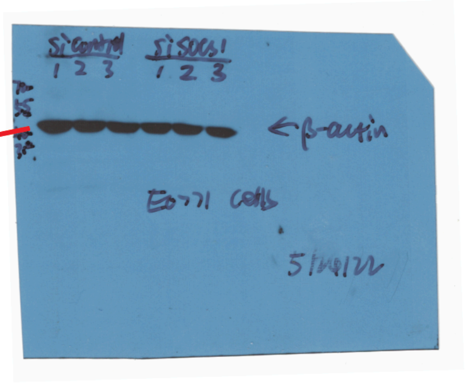

Loading controls for STAT3 and SOCS1 (β-Actin).

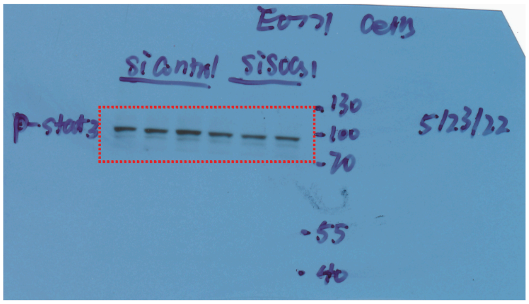

Supplementary Figure 13D, p-STAT3
Cell Signaling Technology (9145), 1:3000

Blot was stripped
and restained with β-Actin.

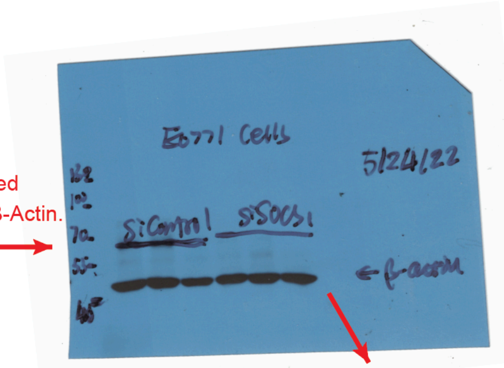

Loading controls for p-STAT3 (β-Actin).
